# Interlaboratory clinical chemistry parameter variation in seven-day acute hydrazine toxicity studies in the Sprague-Dawley rat

**DOI:** 10.1007/s00204-025-04227-5

**Published:** 2025-12-05

**Authors:** Janonna Kadyrov, Samuele Sala, Lucy Grigoroff, Reika Masuda, Samantha Lodge, Timothy M. Ebbels, Michael D. Reily, Donald Robertson, Lois Lehman-McKeeman, John Shockcor, Bruce D. Car, Craig Thomas, John C. Lindon, Julien Wist, Jeremy K. Nicholson, Elaine Holmes

**Affiliations:** 1 Centre for Computational and Systems Medicine, Health Futures Institute, Murdoch University, Perth, WA Australia; 2 Department of Metabolism, Digestion and Reproduction, Faculty of Medicine, Imperial College London, Hammersmith Campus, London, W12 0NN UK; 3 Formerly Pfizer Global R&D, Ann Arbor, MI USA; 4 Formerly Bristol-Myers-Squibb Company, Princeton, NJ USA; 5 Formerly Drug Metabolism and Pharmacokinetics Section, Stine-Haskell Research Center, Dupont Pharmaceuticals Company, Newark, DE USA; 6 ThomaPharma Consulting LLC, McCordsville, IN 46055 USA; 7 Formerly Lilly Research Laboratories, Eli Lilly and Company, Indianapolis, IN 46055 USA; 8 Department of Metabolism, Digestion and Reproduction, Faculty of Medicine, Imperial College London, London, UK; 9 Institute of Global Health Innovation, Faculty of Medicine, Imperial College London, London, SW7 2AZ UK; 10 Chemistry Department, Universidad del Valle, Cali, 76001 Colombia

**Keywords:** Sprague-Dawley, Hydrazine toxicity, Clinical chemistry, Multisite study

## Abstract

Clinical chemistry retains its position as a cornerstone of toxicological assessment, yet inter-laboratory variability in baseline values remains a challenge for the integration and interpretation of multisite datasets. This study leveraged a publicly available clinical chemistry database to assess the impact of inter-laboratory variability in response to hydrazine-induced steatosis. Seventeen clinical chemistry and physico-chemical parameters were evaluated in response to a single dose of hydrazine (at 30 mg/kg or 90 mg/kg) administered to Sprague-Dawley rats (*n* = 83) across five different pharmaceutical companies and compared with sham-dosed control animals. Hydrazine exposure produced a distinct and consistent biochemical signature at 48 h post-dose across the combined sample set from all laboratory sites, characterised by increased serum bilirubin and BUN and decreased serum protein concentrations, alongside atypical reductions in ALT and AST due to transaminase inhibition. Despite sizable inter-laboratory differences in response when considering single assays, multivariate analysis of the complete dataset was able to extract a core pathological response signature. Early changes at 24 h post-dose in AST, ALT, total protein, and calcium demonstrated strong predictive value for 48-h toxicity profiles (AUROC 0.98), underscoring the translational potential of early biomarkers. This study highlights both the robustness and contextual limitations of clinical chemistry data in toxicological studies. It underscores the importance of matched-control designs and multivariate approaches for multisite studies and advocates for the integration of early predictive modelling to optimise study design and align with the principles of the Replace, Reduce, and Refine initiative.

**Supplementary Information:**

The online version contains supplementary material available at 10.1007/s00204-025-04227-5.

## Introduction

The Consortium for Metabonomic Toxicology (COMET) project was an academic-industry collaboration established between Imperial College London and a group of five international pharmaceutical companies to evaluate the utility of metabolic phenotyping approaches in preclinical toxicology (between 2001 and 2007). The project generated a comprehensive dataset of temporal metabolic profiles following exposure to a wide range of reference toxicants with well-defined organ-specific effects (Lindon et al. 2005), focusing on liver and kidney toxicity, which represent two of the most frequent and severe causes of drug attrition with approximately 35–40% of drug candidates being withdrawn due to safety issues (Tosca et al. 2021). Recently, we made the integrated and curated clinical chemistry data publicly available to encourage reuse of this extensive toxicology information on rodent acute toxicity containing 86 toxins and 21 physiological stressors at two different dose levels (Kadyrov et al. 2025; 10.5281/zenodo.14963725).

Clinical chemistry testing in rodent models remains central to nonclinical safety assessment and detection of systemic or localised toxicity. Regulatory agencies like the U.S. Food and Drug Administration (U.S. FDA 1999; U.S. FDA 2007), European Medicines Agency (EMA 2010), and Ministry of Health, Labour and Welfare of Japan (MHLW 1989, 1993, 1999, 2020) have established guidelines recommending standardised clinical pathology panels. However, idiosyncratic toxicities and adverse events continue to challenge drug safety evaluations. Contributing factors to variation in response to drugs include small, single-site studies, limited genetic diversity within test populations and the absence of a single universal standard method for sample collection and preparation and clinical measurements.

A major challenge is the substantial inter-animal variability in rodent toxicity models, driven by minor differences in genetics, metabolism, stress, and animal handling. This biological variability, combined with intra-laboratory differences in instrumentation, reagents, calibration, and technique, potentially limits direct comparisons between laboratories and prevents co-modelling of the data, which impacts statistical power and animal usage. To address this, professional organisations like the American Association for Clinical Chemistry’s Division of Animal Clinical Chemistry (AACC-DACC), the American Society for Veterinary Clinical Pathology (ASVCP) and the Society of Toxicologic Pathologists (STP) have issued harmonised recommendations for clinical pathology testing in nonclinical studies (Weingand et al. 1992; Tomlinson et al. 2013, 2016). Despite considerable efforts to standardise protocols, substantial inter-animal and inter-laboratory variability limits the direct comparability of pre-clinical rodent studies, particularly when toxicity assessments rely on absolute values from single assays (Petterino and Argentino-Storino 2006). Therefore, databases of reference values become more informative when they capture the direction of change from pre-dose baselines and incorporate dynamic responses over an appropriate temporal window.

Here, we utilised a standardised clinical chemistry database generated by the COMET study in combination with a multivariate analytical approach to investigate the influence of intra/inter-animal variability and technical measurement variation on the characterisation of toxic effects. As part of the COMET methodology validation, five pharmaceutical companies conducted a multi-centre study using hydrazine as a model hepatotoxin to induce hepatic steatosis. Single doses of 0 mg/kg, 30 mg/kg, or 90 mg/kg were administered to rats, and a panel of 17 clinical chemistry parameters was measured at 24 h, 48 h, and 168 h post-dose (Lindon et al. 2003).

Hydrazine is known to induce oxidative stress, causing alterations in mitochondrial structure and function, lipid peroxidation, and depletion of glutathione reserves (Scales and Timbrell 1982; Jenner and Timbrell 1994). These pathological changes are reflected in characteristic alterations in both serum, including disrupted lipoprotein and ketone body profiles (Wu et al. 2005), and urine, with increased concentrations of amino acids (Sanins et al. 1992; Keun et al. 2002) with maximum perturbation in metabolic profiles being observed approximately 48 h after dosing at 120 mg/kg (Nicholls et al. 2001).

Here we assess the reproducibility of clinical chemistry data from acute rodent toxicity studies using the COMET harmonised experimental design and protocol in a multi-centre study and benchmark the toxic response against the natural variation in control animals.

## Methods

### Experimental design

Eight- to ten-week-old male Sprague-Dawley rats (*n* = 3455), sourced from Charles River Laboratories, were housed individually in metabolism cages (Harvard Apparatus, Holliston, MA) under standardised light-dark cycles (06:00 to 18:00), temperature (21 ± 2 °C), and humidity (55 ± 10%). Animals were provided Purina 5002 chow and water *ad libitum*. The studies were conducted across five pharmaceutical companies (designated A to E) as part of the Consortium for Metabonomic Toxicology (COMET) project, following a harmonised 7-day acute toxicity protocol (Lindon et al. 2005).

The COMET project conducted a series of acute toxicity studies involving a total of 86 toxins and 21 physiological stressors. Each study consisted of three dose groups: control (a single administration of dose vehicle, typically corn oil or of 0.9% saline), low-dose (at sub-toxic level), and high-dose (acute toxicity/effect), with ten animals per group. Doses were administered at 0 h. Half of the animals in each group were euthanised at 48 h, and the remaining half at 168 h post-dosing. Serum samples were collected at 24 h post-dose and at sacrifice, while urine samples were collected at 16 h pre-dose and at multiple time points post-dose (0, 8, 24, 48, 72, 96, 120, 144, and 168 h). Urine was preserved at 0 ± 2 °C in containers with 1 mL of 1% w/v sodium azide prior to collection, upon which samples were centrifuged (~ 1200 g, 10 min), and stored at −40 °C until analysis.

For the hydrazine study the three dose groups consisted of: control (a single intraperitoneal dose of 0.9% saline), low-dose (30 mg/kg), and high-dose (90 mg/kg) based on expected toxicity responses from previously published studies (Sanins et al. 1992; Nicholls et al. 2001).

Clinical chemistry parameters were measured according to standardised protocols (the full protocol is available in the Supplementary Material of the recently published clinical chemical atlas (Kadyrov et al. 2025) and includes a panel of serum (creatinine, urea nitrogen, alanine aminotransferase (ALT), aspartate aminotransferase (AST), glucose, sodium, potassium, calcium, phosphate, albumin, total protein, total bilirubin) and urine (total volume collected, osmolality, pH, protein and glucose) biomarkers reflecting liver and kidney function. All raw data were deposited in an in-house centralised database.

### Data processing and quality control

All statistical analyses were performed using R Statistical Software (R Core Team 2023), a free and open-source software environment for statistical computing and graphics. A full description of the data curation process has been recently published and is available online (Kadyrov et al. 2025).

### Assessment of inter-laboratory variability

**Fig. 1:**
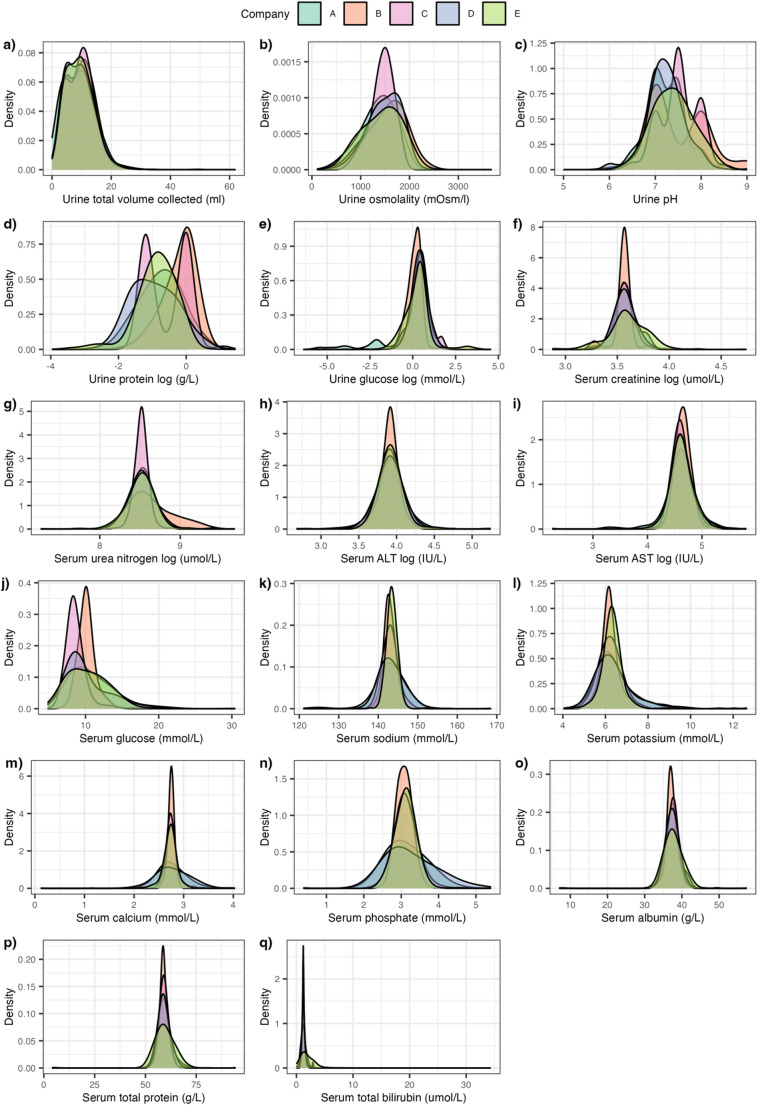
Panel showing distributions of control sample clinical parameters stratified by pharmaceutical company

To evaluate baseline variability across laboratories, the distribution of all clinical chemistry parameters from all control samples and those collected at pre-dose and baseline time points (*n* = 13,085) across five pharmaceutical companies and 113 studies, were stratified by company and visualised as distribution plots (Fig. 1). This provided a broad comparative overview of preclinical background variation, with accompanying summary statistics provided in Supplementary Table S1. All subsequent statistical analyses use samples from time point 24 h, 48 h and 168 h post-dose due to having both a urine and serum sample collection. The mean value for each clinical parameter in the control animals at 24 h, 48 h and 168 h post-dose (*n* = 2834), expressed as the difference in the mean of the individual company compared to the total mean for all companies combined, were tabulated (Supplementary Table S2) and visualised using boxplots (Supplementary Fig. S1).

### Histopathology

Liver tissue samples were fixed in 10% buffered formalin, dehydrated and embedded in paraffin wax, sectioned, stained with haematoxylin and eosin, examined and assessed microscopically. Pathology was assessed by a dedicated veterinary pathologist, and a composite severity score was allocated according to the following scale: (1) no toxicity; (2) minimal toxicity; (3) mild toxicity; (4) moderate toxicity; and (5) severe toxicity (Supplementary Table S3).

### Statistical analysis of clinical chemical parameters

**Fig. 2:**
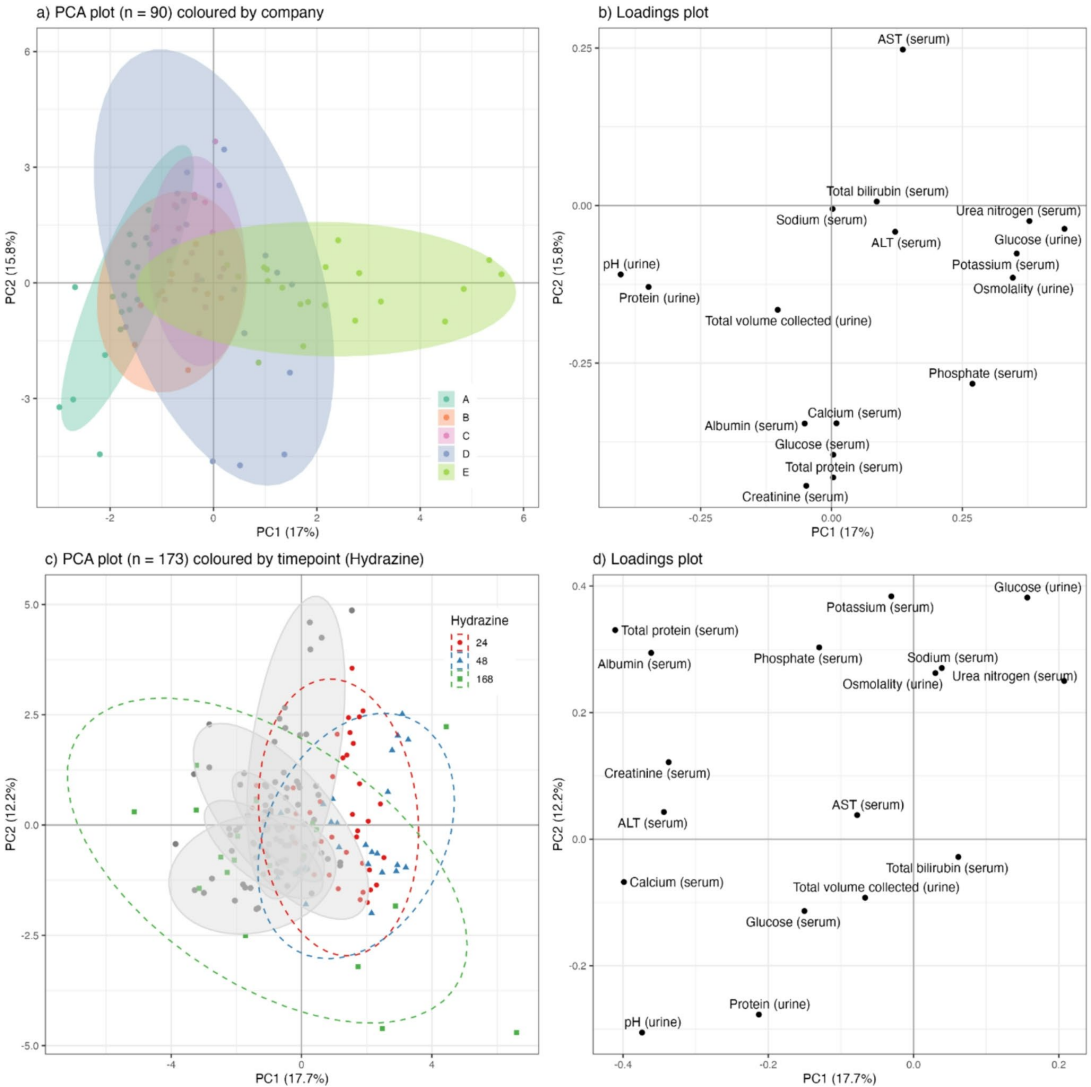
PCA scores and loading plots of the clinical chemical parameters using samples from the hydrazine studies collected at 24 h, 48 h and 168 h post-dose. Figure **a** control samples coloured by company, **b** loadings plots of control samples, **c** control samples with company ellipses shown in grey and hydrazine-dosed samples at 24 h, 48 h and 168 h shown in red, blue and green respectively, **d** loadings plot of control and high-dose samples

**Fig. 3:**
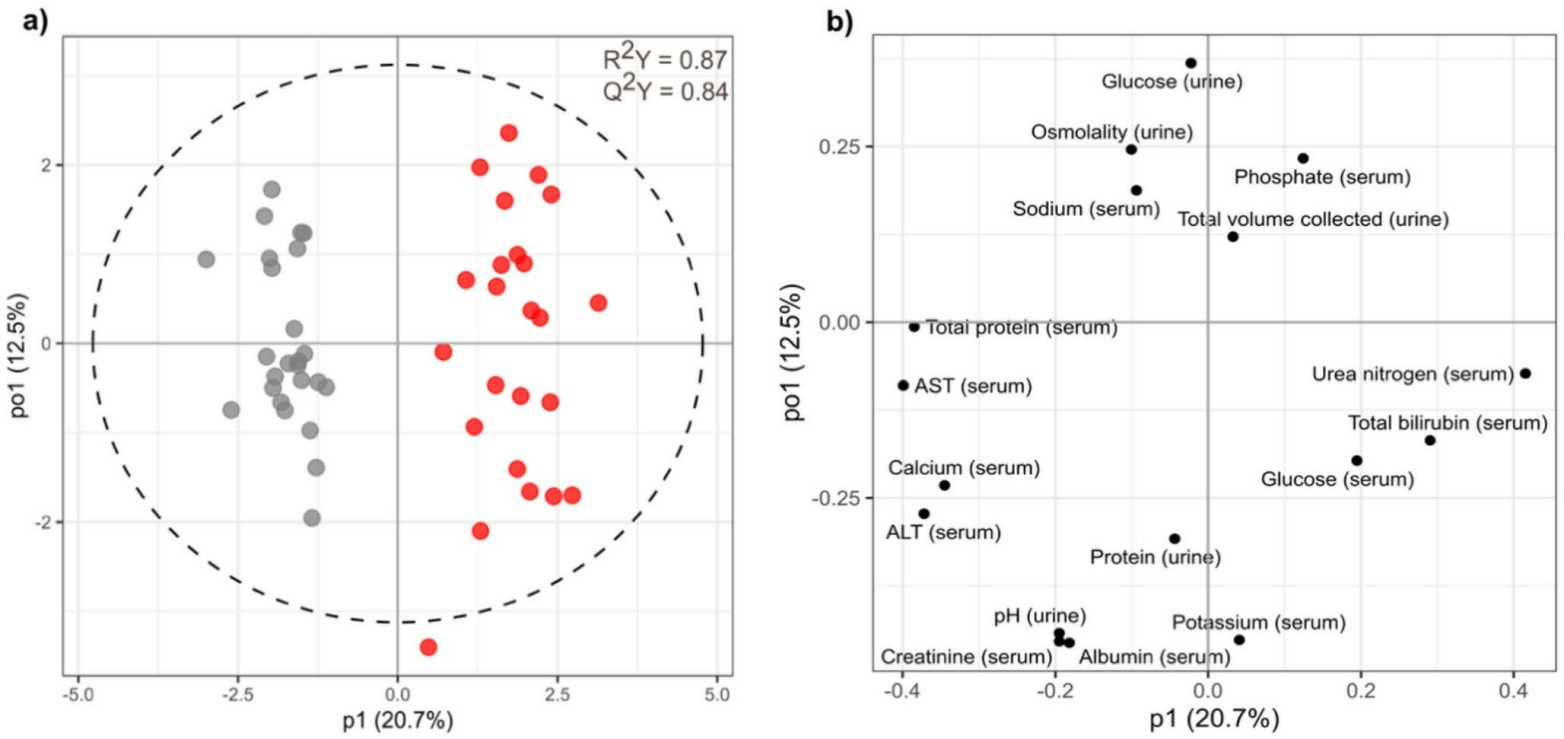
**a** OPLS-DA scores and **b** loadings plots of clinical chemistry parameters comparing control (grey) and high-dose (red) samples from the hydrazine studies at 48 h post-dose, using samples from all companies (*n* = 49)

Principal Component Analysis (PCA) was used to explore variation in the clinical chemistry profiles within and between laboratories. In the first instance, control urine samples at 24 h, 48 h, and 168 h post-dose from only the hydrazine studies (*n* = 90) were analysed (Fig. 2a). Subsequently, a second model was generated using *n* = 2082 control samples collected at 24 h, 48 h, and 168 h post-dose from the full list of 113 toxicity studies (Supplementary Fig. S2a). To evaluate the impact (effect size) of hydrazine dosing against the background of variation (experimental and biological noise) in control animals, PCA models were recalculated with the inclusion of hydrazine high-dose samples at 24 h, 48 h, and 168 h for both the hydrazine-only dataset (*n* = 90 control; *n* = 83 hydrazine-treated) (Fig. 2c) and the total-toxin dataset (*n* = 2082 controls; *n* = 83 hydrazine-treated) (Supplementary Fig. S2c). PCA scores plots were used to visualise the data stratified by company, dose group, and time point. Corresponding loadings plots were generated to identify which clinical parameters contributed most to observed variance for the hydrazine-only dataset (Fig. 2b and d) and the total-toxin dataset (Supplementary Fig. S2b and S2d). PCA is an unsupervised method that reflects the dominant sources of variation in a dataset, which can include experimental noise or inter-site batch effects rather than toxicologically significant signals. Therefore, to more directly evaluate the discriminative power of clinical chemistry profiles for distinguishing high-dose hydrazine effects from baseline variation, supervised multivariate modelling using Orthogonal Projections to Latent Structures Discriminant Analysis (OPLS-DA) was conducted. To identify time points at which hydrazine induced the strongest divergence from control profiles, models were constructed separately for each company and, additionally, for the combined dataset across all companies at 24 h, 48 h, and 168 h post-dose (Fig. 3; Supplementary Fig. S3–S8). The quality of each model was assessed using R^2^Y (explained variance in the response) and Q^2^Y (predictive ability of the model) values for the first predictive component.

### Feature selection and biomarker visualisation

Features contributing most strongly to class separation in the OPLS-DA models were ranked based on their frequency in the top three most discriminating variables across all models (individual companies and the combined dataset) and presented in Supplementary Table S4. To ensure these variables reflected biological responses to hydrazine rather than technical or inter-laboratory noise, each top-ranked feature was cross-checked against the loadings from the earlier PCA models. Features that contributed predominantly to variation in the unsupervised PCA models, especially those separating companies within the control dataset, were flagged as potentially confounded by site-specific experimental effects and interpreted with caution. Additionally, the consistency of feature selection across company-specific OPLS-DA models was compared with the combined (pooled) model. This comparison allowed the assessment of whether the discriminatory variables identified at the individual laboratory level were conserved when all data were aggregated. Features that were most discriminating between control and hydrazine-dosed samples for individual companies at 48 h post-dose were marked with a tick if they were in the top six most discriminating features in their corresponding OPLS-DA model and presented in Table 1.

**Table 1:** Parameters that were in the top 6 most discriminatory features in the OPLS-DA loadings plots comparing hydrazine treated rats with controls 48 h post-dose for each of the 5 companies

Parameter	Company				
	A	B	C	D	E
Serum total protein	✔	✔	✔	✔	✔
Serum calcium	✔	✔	✔	✔	✔
Serum urea nitrogen	✔	✔		✔	✔
Serum total bilirubin		✔	✔	✔	✔
Serum glucose	✔	✔	✔		
Serum AST	✔			✔	
Serum ALT		✔			✔
Serum potassium	✔				✔
Serum phosphate			✔		
Urine pH			✔		
Urine total volume				✔	
Urine protein					✔

**Fig. 4:**
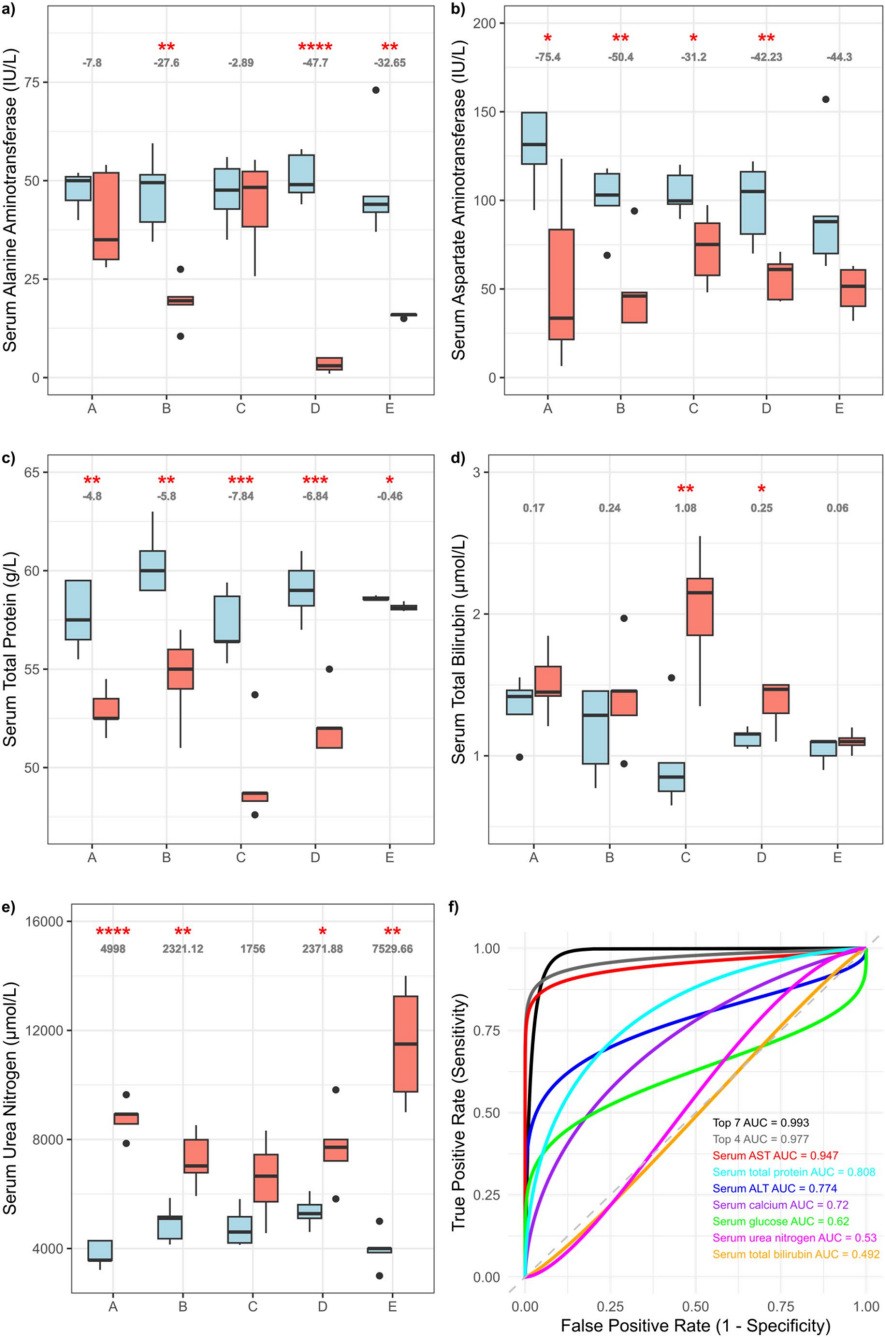
Box and whiskers plots of serum ALT (**a**), AST (**b**), total protein (**c**), total bilirubin (**d**), and urea nitrogen (**e**) values for control (blue) and high-dose (red) samples from hydrazine studies. Slope shown in grey and significance represented as asterisks (*p < 0.05; **p < 0.01; ***p < 0.001; ****p < 0.0001). **f** Comparison of ROC Curves: top 7 clinical chemistry levels at time point 24 h to predict liver histopathology severity score at 48 h post-dose

Box and whisker plots were used to visualise the most consistently discriminatory biomarkers as identified in Supplementary Table S4: ALT (alanine aminotransferase), AST (aspartate aminotransferase), serum total protein, serum bilirubin and BUN (blood urea nitrogen). These plots illustrated trends across time points and companies, both in control and high-dose groups (Fig. 4a–e).

To evaluate the predictive utility of early clinical chemistry changes, an Area Under the Receiver Operating Characteristic (AUROC) curve analysis was conducted using data from 24 h post-dose to predict hepatic histopathology at 48 h post-dose (Fig. 4f). The seven most discriminatory features at 48 h, as identified from the OPLS-DA models (and shown in Table 1), were used as input variables. For each of these seven parameters, a univariate model was generated to assess how well its 24 h value could predict its classification at 48 h (high-dose vs. control). In addition, multivariate models combining the top four and the top seven predictors were created. ROC curves were generated, and AUC values were calculated to evaluate the classification performance of each model.

## Results

### Distributions of standard pre-clinical toxicity study parameters in control rats demonstrate minor differences across laboratory sites

The distribution for each of the measured urine and serum parameters, stratified by pharmaceutical company, was interrogated for the full set of control Sprague-Dawley rats (*n* = 13,085) involved in the COMET project (Fig. 1). The majority of both the urine and serum parameters were normally distributed across the companies. However, some parameters exhibited a left skew (serum glucose and urinary total volume collected), whilst urinary protein demonstrated a bimodal distribution for samples obtained by Company C. An apparent trimodal distribution was observed for Company C and Company B’s urine pH measurement resulting from the use of a urinary dipstick to measure pH, which only provided data rounded to integer values of 1 pH unit in contrast to values obtained using a pH meter with a resolution of 0.01 pH. Ranges and distributions for most clinical parameters showed strong coherence across companies. Although the overall distribution of the values across companies behaves similarly, company differences were observed across parameters, in particular Company C showed differences in concentration ranges for serum urea nitrogen and serum glucose, as well as different distributions for urinary osmolality, protein and pH. Distributions for the serum parameters were better aligned compared to the urinary parameters, consistent with the fact that serum is maintained under tighter homeostatic control compared to urine. Corresponding summary statistics are provided in Supplementary Table S1. These findings demonstrate that, despite minor procedural and instrumentation differences, control clinical chemistry values collected over three years across multiple sites exhibit high concordance. This reinforces the utility of centralised databases for toxicity model development and preclinical toxicology research. The critical question, then, is not whether background variation exists, but whether it meaningfully affects toxicological interpretation. Addressing this question was a primary goal of the subsequent multivariate analyses.

As a test of intra-operability f the COMET protocol, hydrazine was chosen as a test compound due to its ability to elicit a consistent response (Nicholls et al. 2001; Sanins et al. 1992; Timbrell and Harland 1979) with each of the five pharmaceutical companies dosing the compound at the same dose levels following identical protocols. Differences between individual company means from the grand mean in the measured clinical chemical values were visualised through bar plots (Supplementary Fig. S1), and corresponding effect sizes (η^2^) and p-values from ANOVA tests are provided in Supplementary Table S2.

### Toxicological responses to hydrazine are consistent despite control variability

Co-analysis of the 17 urine and plasma clinical chemistry parameters based on the 90 hydrazine study control samples (dose vehicle only) alone, showed that samples from all five centres demonstrated considerable overlap in the PCA scores plot (Fig. 2a), although some centres e.g., Company B produced more tightly clustered data, indicating good biological and analytical reproducibility. The variance in PC1 across the dataset was predominantly driven by serum electrolytes (phosphate, potassium), serum urea nitrogen and urinary glucose, protein and osmolality as evidenced by the model loadings (Fig. 2b). The introduction of samples from rats treated with hydrazine (90 mg/kg), eliciting hepatic steatosis, showed partial clustering of the treated samples in the first principal component, regardless of sample origin i.e. laboratory, especially at 48 h post-dose (Fig. 2c). The introduction of hydrazine treated samples into the model caused an expected convergence of the control samples in the scores plot since the variance attributable to hydrazine (described by PC1) was greater than the variance in the control group (grey coordinates), mainly apparent in PC2 (Fig. 2c). This indicates that the impact of toxicity on variation in clinical parameters is stronger than the baseline variation. Whilst all hydrazine samples were distinct from control samples at 48 h post-dose (as indicated by the separation of hydrazine-dosed samples from the rest of the sample set across the first principal component), some of the samples obtained at either 24 h or 168 h post-dose were overlapped with the control samples, reflecting either later onset of response or early recovery from the toxic insult respectively. The key discriminatory features in this study defining hydrazine toxicity included higher urinary glucose, serum bilirubin, serum urea nitrogen with lower serum total protein, albumin, creatinine, calcium and ALT and lower urine pH in comparison with control samples (Fig. 2d).

The response to hydrazine-induced toxicity at 24 h, 48 h and 168 h post-dose was assessed in the context of the broader control database containing a total of 2082 samples collected from 113 acute toxicity studies and over a three-year period, in order to ascertain the robustness of the multivariate data analysis approach and utility of the biobank of control data (10.5281/zenodo.14963725). This larger control dataset enabled the evaluation of study-specific control groups in a broader physiological context, highlighting the extent of natural biological variability across time and sites. Using this dataset (*n* = 2082 control samples from 24 h, 48 h and 168 h from 113 toxicity studies), the variance in the PCA was dominated by several strong outliers (Supplementary Fig. S2a). Despite this, the inherent pattern in the scores plot included a clear separation between the control samples and hydrazine-dosed samples at 48 h post-dose (Supplementary Fig. S2c), which is consistent with the separation between hydrazine-dosed animals and controls observed when using the control data relating specifically to the hydrazine studies and which related to a single batch of rats maintained under identical environmental conditions. Although, as expected, the dispersion of coordinates relating to the extended control dataset was greater than that observed in the hydrazine study controls alone, this wider variance did not obscure the toxicological signal associated with hydrazine exposure. This highlights that the presence of statistical variation (e.g., the wider spread in the PC scores plots), does not negate or diminish the biological effect of toxicity, which manifests as reproducible shifts in multivariate space. By maintaining this differentiation between natural inter-study variation in control animals and the specific, directional perturbation caused by a toxicant, the biobank appears to serve as a useful context-dependent tool without compromising the detection of genuine toxicological responses.

Since lesion development in acute toxicity studies follows a dynamic trajectory (Gartland et al. 1991), evolving to a maximum pathological effect before resolving over time, the data were stratified by time point and company and analysed using multivariate discriminant analysis (OPLS-DA). The explained and predictive performance metrics (R^2^Y, Q^2^Y) of the model confirm that hydrazine had a strong and reproducible effect on the clinical chemistry parameters across all five companies. Although the response across companies was consistent in terms of the impact on the clinical chemistry parameters, there were evident differences in the time of response. At 24 h, several companies, e.g., Company A, C, D, and E, showed early toxicity signatures (Supplementary Fig. S3 & S4), whereas Company B failed to show distinction between the control and dosed samples at this time point, suggesting delayed response. By 48 h, all companies demonstrated strong separation between control and high-dose samples (Supplementary Fig. S5), with consistent loadings (Supplementary Fig. S6) highlighting shared biological responses to hydrazine. The models generated were of similar strength with predictive power (Q^2^Y) values ranging from 0.79 (company A) to 0.89 (companies C and D). The inter-company similarity in response at 48 h post-dose contrasts with the broader spread seen at 24 h post-dose (Q^2^Y 0.53 company C to 0.92 company D). At 168 h (Supplementary Fig. S7 & S8), the strength of the OPLS-DA models for the recovery trajectories varied from Q^2^Y 0.24 (company B) to 0.75 (company D). Company A and Company C showed convergence of dosed and control samples, consistent with early responders recovering. Conversely, Company B and Company E exhibited strong separation at this later stage, indicative of delayed onset or recovery from toxicity compared to the other laboratory sites. Control and high-dose groups from Company D were consistently differentiated across all time points, indicating enhanced sensitivity of their clinical chemistry measurements to hydrazine exposure, and/or indicating that their hydrazine-induced toxic effects were more persistent. For each company-related model, the six clinical parameters with the strongest weighting were summarised in Table 1.

Histopathological analysis conducted on samples obtained at 48 h and 168 h post-dose indicated that fatty liver, hepatitis, hepatocellular cytoplasmic vacuolation and mononuclear cell infiltrates were present at 48 h (Supplementary Table S3) but were less marked or resolved by 168 h post-dose. Comparing the multivariate clinical chemical scores with the histopathology, the clinical chemistry scores largely reflected the summed histology response (Supplementary Fig. S9 & S10).

### Comparison of hydrazine toxicity clinical chemistry profiles across five pharmaceutical companies

A comparison of high-dose versus control for the combined companies at 48 h post-dose was used to generate a clear set of differential clinical chemistry parameters associated with hydrazine toxicity (Fig. 3). The modulation of enzymes and other measured clinical features following hydrazine toxicity was largely consistent across companies at 48 h post-dose defined by lower levels of serum ALT (Fig. 4a), AST (Fig. 4b), and total protein (Fig. 4c) with higher serum total bilirubin (Fig. 4d) and urea nitrogen (4e). In addition, higher serum glucose and lower serum calcium were also frequently associated with hydrazine toxicity (Supplementary Table S4). The consistent response across study sites reinforces the reliability and robustness of ALT, BUN, total protein and total bilirubin as cross-company biomarkers of hydrazine-induced toxicity, despite minor baseline inter-laboratory variation. Although these parameters consistently emerged as the most consistent discriminators in separating control from high-dose hydrazine-treated animals, company-dependent variation in the rank or weighting of each of these parameters occurred. As expected, the inter-company variation in response to hydrazine was greater at 24 h and 168 h post-dose since early and late responders introduced variability (data not shown). Analysis of control values for each of the 17 measured parameters did not show consistent effect associated with the study run order (aligned to the three-year time frame) but certain batch effects were apparent, for example decreased urine glucose (company E) and serum sodium (company A) was observed for one or two studies and increased serum urea nitrogen values were apparent in five consecutive studies for company B (Supplementary Fig. S11).

### Multi-component clinical chemistry profiles at 24 h and 48 h reflect hepatic histopathology

To assess whether the multiparameter clinical chemistry profiles at 24 h post-dose could serve as predictors of extent of liver pathology, the top seven discriminatory biomarkers extracted from the 48 h OPLS-DA models (as per Table 1) were tested as to whether their 24 h profiles could classify animals into control or high-dose groups at 48 h. The combined seven parameter 24 h model demonstrated excellent predictive power for toxic response at 48 h post-dose as shown by the AUROC curve (0.99) in Fig. 4f, which outperformed each biomarker when analysed individually, wherein the AUROC values ranged from 0.49 (serum bilirubin) to 0.95 (serum AST). Thus, the AUROC curve indicated strong predictive capacity even at early time points. This result suggests that the combination of several early biomarkers may be sufficient for anticipating downstream toxicological effects. It should be noted that although AST did not rank as the top discriminatory parameter for any company at 48 h, it was the strongest feature in the combined OPLS-DA model at 48 h due to its consistent behaviour across all laboratory sites (Supplementary Table S4) and was also the strongest parameter in the ROC curve analysis (Fig. 4f). The increase in the AUROC values only gains a small increment when comparing AST alone (0.95) to the seven-parameter value (0.99). However, the inclusion of the multiparameter signature as opposed to relying on individual parameters, proved more resilient to inter-laboratory variation and provides a more robust and comprehensive reflection of systemic toxicity signatures.

## Discussion

This large-scale study re-enforces the well-established robustness of clinical chemistry parameters in detecting systemic toxicity, despite minor site-specific variation in baseline values. Using data extracted from a publicly available clinical chemistry atlas for a five-site rodent toxicology study exploring hydrazine as a model for steatosis (Kadyrov et al. 2025, 10.5281/zenodo.14963725), we demonstrated that differences in clinical chemical parameters attributable to normal physiological variation are not a barrier to detecting consistent toxicological signatures, as illustrated for hydrazine at a dose level that reproducibly induces steatosis. The use of a pre-existing clinical chemistry database resource aligns with the shift towards alternative, human-relevant predictive models in alignment with the 3Rs (Replacement, Reduction, and Refinement of animal use) (NC3Rs 2025).

Initial inspection of 17 clinical chemistry and physico-chemical parameters for control samples, collected over a three-year period from five different pharmaceutical organisations, indicated a largely stable baseline. However, a key observation was the variability in some clinical chemistry parameters across laboratory sites, which was particularly apparent in control animals. Although each Pharmaceutical Company adhered to standardised regulatory protocols, the baseline values for several analytes differed both in magnitude and distribution. This inter-laboratory variability likely arises from a combination of factors, including differences in analytical platforms, reagent batches, staff practices, and animal sourcing. The presence of distinct site-specific “fingerprints” in control profiles highlights the importance of contextual normalisation or matched design strategies when comparing treatment effects across multisite studies. Notably, some companies exhibited higher within-group variance, which, despite adherence to standardised protocols, may reflect subtle, residual influences such as strain-specific responses, undetected microbiome variation, differences in local environmental conditions, or operational variance. While these factors are tightly controlled in regulatory studies, minor inter-site differences can persist even under highly controlled conditions, e.g., due to variations in animal handling, local water quality, or facility-specific microbial flora (Ericsson and Franklin 2021; Rohde et al. 2007; Bidot et al. 2018; Robosky et al. 2005; Ericsson et al. 2015; Nicholson et al. 2002). While the potential for drift in host or microbial genomes over the three-year period cannot be entirely excluded, our data did not reveal any clear correlation between study timing and the observed inter-batch variability. This suggests that temporal factors alone are unlikely to account for the batch differences, although subtle, cumulative effects cannot be ruled out without deeper longitudinal or genomic analysis. While such variability did not obscure the overall comparability of data within individual sites, it introduced significant challenges for pooled, multi-site analyses. This observation is of great experimental significance, particularly in academic toxicology studies. Individual laboratories were consistently able to detect toxicological signals within their own datasets, showing robust local interpretation. However, when data were combined across sites, inter-laboratory differences in baseline values and measurement practices became more pronounced, potentially confounding statistical models. These findings do not challenge the current regulatory or industrial practices, which generally rely on within-study consistency, but rather, highlight that inter-laboratory data harmonisation is difficult to achieve in practice. While minor variations between companies were observed, these variations fell within expected physiological ranges, as reported previously in the Rodent Clinical Chemistry Atlas (Kadyrov et al. 2025) and by Petterino and Argentino-Storino (2006).

When the hydrazine-treated Sprague-Dawley samples were included in the analysis using the full control rat dataset (*n* = 2082 controls; *n* = 83 hydrazine-treated), a degree of separation from the controls was observed. However, this signal was partially masked by the broad variation among the control samples, collected over an extended time frame and from multiple sources. As a result, global models integrating data across all sites captured only a fraction of the hydrazine-induced perturbations, with many treated samples falling within the multivariate distribution of untreated animals. Additionally, interpretation of the global statistical loadings plots proved challenging, as the diffuse and inconsistent contributions of variables limited mechanistic inference. Taking a meta-analysis approach using a matched design whereby hydrazine-treated rats were compared to their corresponding controls sampled contemporaneously under identical laboratory conditions, the impact of hydrazine treatment on clinical chemistry parameters became more distinct. Although inter-site differences in control profiles persisted, they were constrained within the natural envelope of each site’s physiological variability. Within this bounded variation, each laboratory’s control cohort occupied a distinct position in multivariate space, possibly shaped by subtle, cumulative differences in animal cohorts and husbandry practices.

Further analysis of the pair-matched controls and hydrazine treated samples using OPLS-DA yielded models with strong discriminatory power for hydrazine toxicity for all companies at 48 h post-dose, aligned with histopathological findings, underscoring the toxicological relevance of the observed clinical chemistry changes. Although the hydrazine-treated group was distinct from control based on the data from each company, modelled individually, the parameters driving the toxin-related response were not entirely homogeneous across companies. For example, increased serum urea nitrogen was the strongest discriminator at 48 h post-dose for company A whilst for company D, decreased serum ALT held equal weighting. Despite variation in the specific variables contributing to each model, certain analytes were repeatedly identified across sites, as summarised in Supplementary Table 4. This recurring inclusion of key parameters suggests that, while laboratory-specific factors shape the overall profile, a subset of biomarkers reliably captures the hepatotoxic effects of hydrazine exposure. These findings highlight both the value and the limitations of clinical chemistry as a translational tool: although sensitive to toxic insults, its interpretability is highly context-dependent, particularly in multisite settings where baseline variability must be carefully managed. This is reinforced in the box and whiskers plots where total bilirubin was strongly discriminatory for companies C and D at 48 h post-dose, but was not significantly different for the other three companies at the same time point.

Hydrazine is a potent hepatotoxic agent that induces oxidative stress, mitochondrial dysfunction, and lipid peroxidation, particularly targeting hepatocytes (Hussain and Frazier 2002). The changes observed in a rat model of acute hydrazine toxicity reflect the progression of underlying pathological mechanisms. A single dose of hydrazine at this level has been shown to induce a toxic response that typically peaks around 48 h post-administration, after which the tissue lesions begin to resolve, and serum biochemistry gradually returns to baseline or pre-dose levels (Nicholls et al. 2001; Garrod et al. 2005; Sanins et al. 1992). Here, we also observe maximum impact at 48 h post-dose. Hydrazine elicited a classical response to hepatotoxic insult in terms of increased serum total bilirubin, indicating impaired hepatic clearance or cholestasis (both hallmarks of hepatotoxicity) and decreased serum protein, likely reflecting compromised hepatic synthetic function or altered protein metabolism. The liver is the primary site of albumin and globulin protein synthesis. Acute hydrazine toxicity impairs protein synthesis by damaging hepatocytes and disrupting transcriptional and translational machinery. In acute settings, this may manifest as hypoproteinemia, particularly hypoalbuminemia, reflecting reduced synthetic capacity or hepatic dysfunction. Other features of the clinical profile were toxin specific rather than organ specific. For example, the consistent depletion of enzymes ALT and AST in the serum of hydrazine-treated rats reflects the transaminase-inhibiting effects of hydrazine (Sanins et al. 1992; Garrod et al. 2005). This contrasts with most liver toxins where the classical response is an elevation in these liver enzymes (Waterfield et al. 1993). The differential behaviour of ALT and AST here underscores the importance of understanding drug-specific mechanisms. The levels of AST and ALT are thus unreliable measures of liver toxicity in the presence of hydrazine or indeed other compounds that act as transaminase inhibitors such as drugs like hydralazine (Timbrell and Harland 1979).

Compared to univariate analysis, the multivariate approach provided a more powerful and holistic means of detecting the toxicological signature of hydrazine exposure. While individual clinical chemistry parameters exhibited modest or variable changes when assessed in isolation, these alterations often fell within the range of normal physiological variation, especially when considering inter-laboratory differences. Univariate comparisons, therefore, lacked the sensitivity to detect subtle but coordinated shifts in biochemical homeostasis induced by hydrazine. In contrast, multivariate techniques, such as principal component analysis (PCA) and orthogonal projections to latent structures discriminant analysis (OPLS-DA), leveraged the covariance structure among multiple analytes to identify consistent patterns of perturbation. This systems-level perspective enabled the extraction of latent variables that more effectively distinguished treated from control animals, even in the presence of substantial background variability. The strength of this approach was particularly evident in the matched-control OPLS-DA models, where multivariate discrimination aligned closely with histopathological outcomes and revealed treatment effects that would have been obscured by univariate noise.

Using the OPLS-DA models to identify parameters that clearly differentiated control from hydrazine-treated rats, ROC curves were built based on the 24 h post-dose time point to ascertain whether these profiles were predictive of the clinical chemistry profiles at 48 h post-dose. Whereas some of the parameters that dominated the 24 h post-dose toxicity profiles were not predictive of the response at 48 h post-dose (serum glucose, serum urea nitrogen and serum bilirubin), decreased concentrations of serum AST, ALT, total protein and calcium at 24 h post-dose produced an AUROC curve (0.98) that was predictive of hydrazine toxicity at 48 h post-dose. The ability to use 24 h clinical chemistry data to predict 48 h toxicity profiles highlights the translational relevance of early biomarkers. This approach, illustrated in Fig. 4f, underscores the potential of leveraging early biological responses for forecasting downstream effects and optimising study design in toxicology. It also supports a future direction for incorporating predictive modelling into early preclinical toxicology workflows.

## Conclusion

Our evaluation of multi-centre clinical chemistry data using hydrazine toxicity models affirms that standardised clinical pathology measurements retain their power as tools for preclinical toxicity assessment. Despite minor baseline inter-laboratory differences attributable to factors such as animal husbandry, environmental differences and analytical platform variability, multivariate approaches like PCA and OPLS-DA were able to extract a core signature of the steatotic toxicity ensuring that meaningful biological interpretation was possible. Furthermore, the ability to predict 48 h toxicity profiles using 24 h clinical chemistry data demonstrates the prognostic utility of early biomarker signatures. This study emphasises the need for context-aware data interpretation strategies in multisite studies and signposts the need for generation of deeper phenotypic datasets to enhance our understanding of individual susceptibility to xenobiotics and reduce the incidence of severe adverse drug reactions.

## Supplementary Information

Below is the link to the electronic supplementary material.

Supplementary Material 1

## Data Availability

The datasets analysed during the current study are available in the zenodo.org repository, 10.5281/zenodo.14963725.
