# Supplementary material for: Interlaboratory clinical chemistry parameter variation in seven-day acute hydrazine toxicity studies in the Sprague-Dawley rat
Source: Arch Toxicol. 2025 Dec 5;100(3):917–30. doi: 10.1007/s00204-025-04227-5 (PMC12967443; doi:10.1007/s00204-025-04227-5)
Supplement: Supplementary file 1 — Supplementary Material 1 [file 204_2025_4227_MOESM1_ESM.docx]

Interlaboratory clinical chemistry parameter variation in seven-day acute hydrazine toxicity studies in the Sprague-Dawley rat

Janonna Kadyrov^1,2^, Samuele Sala^1,2^, Lucy Grigoroff^1,2^, Reika Masuda^1,2^, Samantha Lodge^1,2^, Timothy M. Ebbels^3^, Michael D. Reily^4^, Donald Robertson^4^, Lois Lehman-McKeeman^5^, John Shockcor^6^, Bruce D. Car^5^, Craig Thomas^7^, John C. Lindon^8^, Julien Wist^1,2,9,10^*, Jeremy K. Nicholson^2,9^*, Elaine Holmes^1,2,8^*

*^1^ Australian National Phenome Centre, Health Futures Institute, Murdoch University, Perth, WA, Australia*

*^2^ Centre for Computational and Systems Medicine, Health Futures Institute, Murdoch University, Perth, WA, Australia*

*^3^ Department of Metabolism, Digestion and Reproduction, Faculty of Medicine, Hammersmith Campus, Imperial College London, London W12 0NN, UK*

*^4^ Formerly Pfizer Global R&D, Ann Arbor, MI, USA*

*^5^ Formerly Bristol-Myers-Squibb Company, Princeton, NJ, USA*

*^6^ Formerly Drug Metabolism and Pharmacokinetics Section, Dupont Pharmaceuticals Company, Stine-Haskell Research Center, Newark, Delaware, USA*

*^7^ThomaPharma Consulting LLC, McCordsville, IN 46055; Formerly Lilly Research Laboratories, Eli Lilly and Company, Indianapolis, Indiana, United States of America.*

*^8^ Department of Metabolism, Digestion and Reproduction, Faculty of Medicine, Imperial College London, London, UK*

*^9^ Institute of Global Health Innovation, Faculty of Medicine, Imperial College London, London SW7 2AZ, UK*

*^10^ Chemistry Department, Universidad del Valle, 76001 Cali, Colombia*

**Supplementary Material**

**Supplementary Table S1** Summary statistics of control samples stratified by pharmaceutical company for each clinical chemical parameter

| **Parameter** | **Company** | **Median (min - max)** | **Mean (± SD)** |
| --- | --- | --- | --- |
| Urine Total Volume Collected (mL) | A | 8.8 (0 - 42) | 8.82 (± 5.14) |
|  | B | 9.8 (0.8 - 39.4) | 9.69 (± 4.71) |
|  | C | 10 (0.8 - 62) | 9.46 (± 4.76) |
|  | D | 9 (1 - 32) | 9.28 (± 4.72) |
|  | E | 9 (1 - 50) | 9.39 (± 4.83) |
| Urine Osmolality (mOsm/L) | A | 1400 (101 - 2175) | 1358.55 (± 362.75) |
|  | B | 1586 (217 - 3684) | 1535.59 (± 408.53) |
|  | C | 1469.36 (644.87 - 2000.6) | 1439.11 (± 241.52) |
|  | D | 1521 (220 - 2416) | 1490.02 (± 336.67) |
|  | E | 1464 (277 - 2518) | 1431.08 (± 404.85) |
| Urine pH | A | 7.2 (5.95 - 8.5) | 7.24 (± 0.42) |
|  | B | 7.2 (6 - 9) | 7.49 (± 0.62) |
|  | C | 7.5 (6 - 9) | 7.52 (± 0.41) |
|  | D | 7.22 (5 - 9) | 7.23 (± 0.41) |
|  | E | 7.4 (6 - 8.7) | 7.39 (± 0.45) |
| Urine Protein (g/L) | A | 0.49 (0.03 - 3.1) | 0.58 (± 0.39) |
|  | B | 0.9 (0 - 3.51) | 0.91 (± 0.41) |
|  | C | 0.58 (0 - 3.2) | 0.68 (± 0.43) |
|  | D | 0.37 (0.05 - 4.3) | 0.5 (± 0.39) |
|  | E | 0.43 (0 - 2.61) | 0.49 (± 0.26) |
| Urine Glucose (mmol/L) | A | 1.4 (0 - 15) | 1.46 (± 1.04) |
|  | B | 1.15 (0 - 8.48) | 1.18 (± 0.54) |
|  | C | 1.56 (0.06 - 96.79) | 1.82 (± 2.19) |
|  | D | 1.5 (0.2 - 11.4) | 1.7 (± 0.94) |
|  | E | 1.38 (0 - 80.26) | 2.09 (± 4.57) |
| Serum Creatinine (umol/L) | A | 35.36 (26.36 - 53.04) | 36.12 (± 4.13) |
|  | B | 35.36 (26.36 - 44.2) | 35.1 (± 2.69) |
|  | C | 35.36 (25.36 - 73.36) | 35.61 (± 4.33) |
|  | D | 35.36 (28.36 - 46.36) | 35.81 (± 3.44) |
|  | E | 35.36 (0 - 114.92) | 37.01 (± 7.5) |
| Serum Urea Nitrogen (umol/L) | A | 5000 (2210 - 8000) | 5030.69 (± 781.53) |
|  | B | 5249.2 (2917.4 - 11010) | 5849.31 (± 1684.36) |
|  | C | 5001.15 (2415 - 7465) | 5006.22 (± 447.84) |
|  | D | 5000 (2500 - 8959.53) | 5093.68 (± 817.38) |
|  | E | 5000 (1430 - 16000) | 5114.92 (± 1204.73) |
| Serum Alanine Aminotransferase (IU/L) | A | 50 (16.5 - 190) | 51.25 (± 13.03) |
|  | B | 50.21 (32.5 - 115) | 50.21 (± 6.66) |
|  | C | 50 (28.1 - 71.9) | 50.4 (± 7.72) |
|  | D | 50 (21 - 84) | 50.63 (± 8.55) |
|  | E | 50.25 (14.5 - 177.5) | 51.35 (± 10.95) |
| Serum Aspartate Aminotransferase (IU/L) | A | 100.25 (25.5 - 271) | 104.65 (± 29.44) |
|  | B | 104.5 (69 - 215.5) | 105.7 (± 15.61) |
|  | C | 100 (58.6 - 187.6) | 103.28 (± 18.71) |
|  | D | 100 (52 - 336) | 105.46 (± 25.24) |
|  | E | 100.25 (9.5 - 198.5) | 104.2 (± 21.75) |
| Serum Glucose (mmol/L) | A | 9.95 (4.8 - 30.47) | 10.87 (± 3.13) |
|  | B | 10.02 (6.71 - 20.04) | 10.2 (± 1.36) |
|  | C | 8.4 (5.4 - 14.9) | 8.58 (± 1.28) |
|  | D | 9.03 (5.1 - 22.7) | 10.09 (± 2.85) |
|  | E | 10.05 (5.55 - 19.15) | 10.25 (± 2.56) |
| Serum Sodium (mmol/L) | A | 143 (121 - 168.55) | 142.96 (± 4.38) |
|  | B | 143 (138 - 150.5) | 142.89 (± 1.42) |
|  | C | 143.02 (135.75 - 157.65) | 143.05 (± 2.13) |
|  | D | 143 (121 - 155) | 143.39 (± 3.19) |
|  | E | 143.34 (138 - 152) | 143.45 (± 1.37) |
| Serum Potassium (mmol/L) | A | 6.2 (4.03 - 11.32) | 6.38 (± 0.89) |
|  | B | 6.2 (5 - 8.35) | 6.22 (± 0.43) |
|  | C | 6.2 (4.84 - 7.8) | 6.22 (± 0.52) |
|  | D | 6.2 (4.5 - 10.05) | 6.38 (± 0.88) |
|  | E | 6.3 (4.55 - 12.65) | 6.35 (± 0.7) |
| Serum Calcium (mmol/L) | A | 2.73 (2.08 - 4.04) | 2.77 (± 0.29) |
|  | B | 2.74 (2.54 - 2.94) | 2.75 (± 0.06) |
|  | C | 2.74 (1.15 - 3.4) | 2.74 (± 0.16) |
|  | D | 2.74 (1.76 - 3.72) | 2.78 (± 0.32) |
|  | E | 2.74 (0.12 - 3.39) | 2.72 (± 0.19) |
| Serum Phosphate (mmol/L) | A | 3.11 (2.07 - 5.31) | 3.15 (± 0.53) |
|  | B | 3.09 (2.46 - 4.01) | 3.08 (± 0.2) |
|  | C | 3.11 (2.17 - 4.29) | 3.12 (± 0.31) |
|  | D | 3.11 (1.81 - 5.41) | 3.25 (± 0.66) |
|  | E | 3.11 (0.38 - 4.64) | 3.11 (± 0.31) |
| Serum Albumin (g/L) | A | 37.45 (32.95 - 49.45) | 37.64 (± 2.01) |
|  | B | 37.17 (32.45 - 42.45) | 37.28 (± 1.43) |
|  | C | 37.45 (30.65 - 57.45) | 37.34 (± 1.9) |
|  | D | 37.45 (31.45 - 43.09) | 37.43 (± 1.75) |
|  | E | 37.45 (6.95 - 46.95) | 37.53 (± 3.13) |
| Serum Total Protein (g/L) | A | 59 (51 - 71) | 59.29 (± 3.16) |
|  | B | 59 (52 - 68.5) | 58.97 (± 2.2) |
|  | C | 59 (47.35 - 94.35) | 59.06 (± 2.78) |
|  | D | 59 (50 - 68.15) | 59.16 (± 2.76) |
|  | E | 59 (4 - 80) | 59.35 (± 5.9) |
| Serum Total Bilirubin (umol/L) | A | 1.2 (0.69 - 5.5) | 1.32 (± 0.42) |
|  | B | 1.2 (0.01 - 3.42) | 1.26 (± 0.4) |
|  | C | 1.2 (0.1 - 2.65) | 1.24 (± 0.4) |
|  | D | 1.2 (0 - 2.1) | 1.21 (± 0.27) |
|  | E | 1.2 (0.34 - 34.34) | 2.11 (± 2.27) |


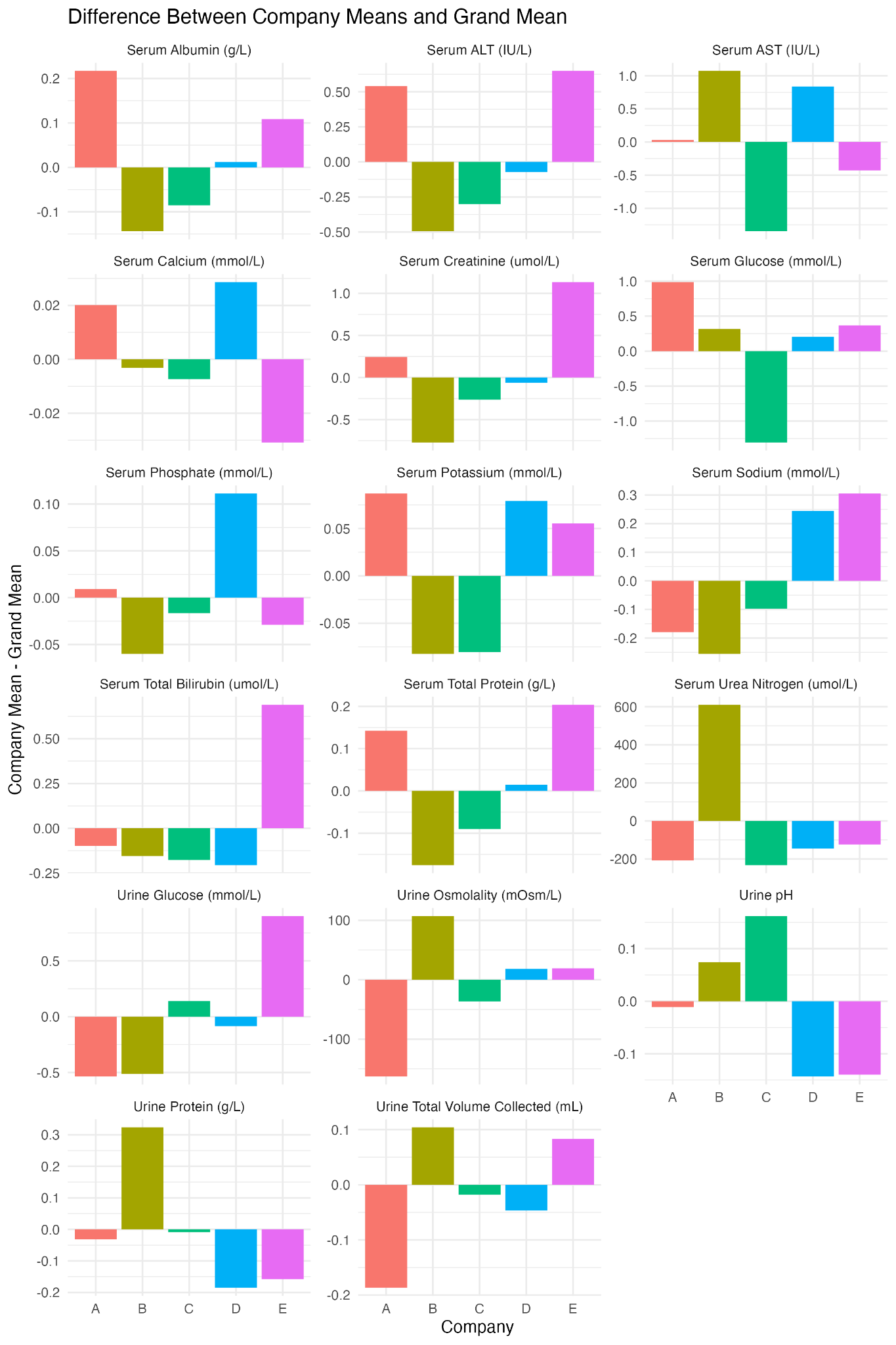


**Supplementary Fig. S1** Boxplot showing the difference between individual company means and the grand mean for each clinical chemical parameter using control samples taken at 24 h, 48 h and 168 h post-dose

**Supplementary Table S2** Comparability of clinical chemical parameters across companies using control samples taken at 24 h, 48 h and 168 h post-dose

| Parameter | Company | Company mean | Grand mean | Mean difference | Eta-squared (η²) | p Value F-Test |
| --- | --- | --- | --- | --- | --- | --- |
| Serum ALT (IU/L) | A | 51.247 | 50.706 | 0.541 | 0.002 | 0.302 |
| Serum ALT (IU/L) | B | 50.211 | 50.706 | -0.494 | 0.002 | 0.302 |
| Serum ALT (IU/L) | C | 50.405 | 50.706 | -0.301 | 0.002 | 0.302 |
| Serum ALT (IU/L) | D | 50.634 | 50.706 | -0.072 | 0.002 | 0.302 |
| Serum ALT (IU/L) | E | 51.355 | 50.706 | 0.649 | 0.002 | 0.302 |
| Serum AST (IU/L) | A | 104.654 | 104.624 | 0.030 | 0.002 | 0.430 |
| Serum AST (IU/L) | B | 105.698 | 104.624 | 1.073 | 0.002 | 0.430 |
| Serum AST (IU/L) | C | 103.278 | 104.624 | -1.346 | 0.002 | 0.430 |
| Serum AST (IU/L) | D | 105.461 | 104.624 | 0.837 | 0.002 | 0.430 |
| Serum AST (IU/L) | E | 104.195 | 104.624 | -0.429 | 0.002 | 0.430 |
| Serum Albumin (g/L) | A | 37.639 | 37.422 | 0.217 | 0.003 | 0.123 |
| Serum Albumin (g/L) | B | 37.278 | 37.422 | -0.144 | 0.003 | 0.123 |
| Serum Albumin (g/L) | C | 37.336 | 37.422 | -0.085 | 0.003 | 0.123 |
| Serum Albumin (g/L) | D | 37.434 | 37.422 | 0.012 | 0.003 | 0.123 |
| Serum Albumin (g/L) | E | 37.531 | 37.422 | 0.109 | 0.003 | 0.123 |
| Serum Calcium (mmol/L) | A | 2.770 | 2.750 | 0.020 | 0.009 | 0.001 |
| Serum Calcium (mmol/L) | B | 2.747 | 2.750 | -0.003 | 0.009 | 0.001 |
| Serum Calcium (mmol/L) | C | 2.743 | 2.750 | -0.007 | 0.009 | 0.001 |
| Serum Calcium (mmol/L) | D | 2.779 | 2.750 | 0.029 | 0.009 | 0.001 |
| Serum Calcium (mmol/L) | E | 2.719 | 2.750 | -0.031 | 0.009 | 0.001 |
| Serum Creatinine (umol/L) | A | 36.117 | 35.872 | 0.244 | 0.018 | <0.001 |
| Serum Creatinine (umol/L) | B | 35.100 | 35.872 | -0.772 | 0.018 | <0.001 |
| Serum Creatinine (umol/L) | C | 35.610 | 35.872 | -0.263 | 0.018 | <0.001 |
| Serum Creatinine (umol/L) | D | 35.810 | 35.872 | -0.063 | 0.018 | <0.001 |
| Serum Creatinine (umol/L) | E | 37.006 | 35.872 | 1.133 | 0.018 | <0.001 |
| Serum Glucose (mmol/L) | A | 10.872 | 9.886 | 0.986 | 0.108 | <0.001 |
| Serum Glucose (mmol/L) | B | 10.203 | 9.886 | 0.317 | 0.108 | <0.001 |
| Serum Glucose (mmol/L) | C | 8.580 | 9.886 | -1.306 | 0.108 | <0.001 |
| Serum Glucose (mmol/L) | D | 10.091 | 9.886 | 0.204 | 0.108 | <0.001 |
| Serum Glucose (mmol/L) | E | 10.254 | 9.886 | 0.368 | 0.108 | <0.001 |
| Serum Phosphate (mmol/L) | A | 3.149 | 3.140 | 0.009 | 0.020 | <0.001 |
| Serum Phosphate (mmol/L) | B | 3.080 | 3.140 | -0.060 | 0.020 | <0.001 |
| Serum Phosphate (mmol/L) | C | 3.124 | 3.140 | -0.017 | 0.020 | <0.001 |
| Serum Phosphate (mmol/L) | D | 3.252 | 3.140 | 0.111 | 0.020 | <0.001 |
| Serum Phosphate (mmol/L) | E | 3.111 | 3.140 | -0.029 | 0.020 | <0.001 |
| Serum Potassium (mmol/L) | A | 6.385 | 6.298 | 0.087 | 0.013 | <0.001 |
| Serum Potassium (mmol/L) | B | 6.215 | 6.298 | -0.082 | 0.013 | <0.001 |
| Serum Potassium (mmol/L) | C | 6.217 | 6.298 | -0.080 | 0.013 | <0.001 |
| Serum Potassium (mmol/L) | D | 6.377 | 6.298 | 0.079 | 0.013 | <0.001 |
| Serum Potassium (mmol/L) | E | 6.353 | 6.298 | 0.055 | 0.013 | <0.001 |
| Serum Sodium (mmol/L) | A | 142.965 | 143.144 | -0.180 | 0.008 | 0.003 |
| Serum Sodium (mmol/L) | B | 142.889 | 143.144 | -0.255 | 0.008 | 0.003 |
| Serum Sodium (mmol/L) | C | 143.047 | 143.144 | -0.097 | 0.008 | 0.003 |
| Serum Sodium (mmol/L) | D | 143.388 | 143.144 | 0.244 | 0.008 | 0.003 |
| Serum Sodium (mmol/L) | E | 143.450 | 143.144 | 0.305 | 0.008 | 0.003 |
| Serum Total Bilirubin (umol/L) | A | 1.319 | 1.417 | -0.098 | 0.094 | <0.001 |
| Serum Total Bilirubin (umol/L) | B | 1.262 | 1.417 | -0.155 | 0.094 | <0.001 |
| Serum Total Bilirubin (umol/L) | C | 1.240 | 1.417 | -0.176 | 0.094 | <0.001 |
| Serum Total Bilirubin (umol/L) | D | 1.211 | 1.417 | -0.205 | 0.094 | <0.001 |
| Serum Total Bilirubin (umol/L) | E | 2.106 | 1.417 | 0.689 | 0.094 | <0.001 |
| Serum Total Protein (g/L) | A | 59.288 | 59.146 | 0.142 | 0.002 | 0.511 |
| Serum Total Protein (g/L) | B | 58.970 | 59.146 | -0.175 | 0.002 | 0.511 |
| Serum Total Protein (g/L) | C | 59.056 | 59.146 | -0.090 | 0.002 | 0.511 |
| Serum Total Protein (g/L) | D | 59.160 | 59.146 | 0.015 | 0.002 | 0.511 |
| Serum Total Protein (g/L) | E | 59.350 | 59.146 | 0.204 | 0.002 | 0.511 |
| Serum Urea Nitrogen (umol/L) | A | 5,030.690 | 5,238.869 | -208.179 | 0.086 | <0.001 |
| Serum Urea Nitrogen (umol/L) | B | 5,849.309 | 5,238.869 | 610.440 | 0.086 | <0.001 |
| Serum Urea Nitrogen (umol/L) | C | 5,006.224 | 5,238.869 | -232.645 | 0.086 | <0.001 |
| Serum Urea Nitrogen (umol/L) | D | 5,093.682 | 5,238.869 | -145.187 | 0.086 | <0.001 |
| Serum Urea Nitrogen (umol/L) | E | 5,114.919 | 5,238.869 | -123.950 | 0.086 | <0.001 |
| Urine Glucose (mmol/L) | A | 1.326 | 1.862 | -0.535 | 0.035 | <0.001 |
| Urine Glucose (mmol/L) | B | 1.349 | 1.862 | -0.513 | 0.035 | <0.001 |
| Urine Glucose (mmol/L) | C | 2.002 | 1.862 | 0.140 | 0.035 | <0.001 |
| Urine Glucose (mmol/L) | D | 1.777 | 1.862 | -0.085 | 0.035 | <0.001 |
| Urine Glucose (mmol/L) | E | 2.763 | 1.862 | 0.902 | 0.035 | <0.001 |
| Urine Osmolality (mOsm/L) | A | 1,429.359 | 1,592.067 | -162.708 | 0.071 | <0.001 |
| Urine Osmolality (mOsm/L) | B | 1,699.294 | 1,592.067 | 107.228 | 0.071 | <0.001 |
| Urine Osmolality (mOsm/L) | C | 1,555.503 | 1,592.067 | -36.564 | 0.071 | <0.001 |
| Urine Osmolality (mOsm/L) | D | 1,610.384 | 1,592.067 | 18.317 | 0.071 | <0.001 |
| Urine Osmolality (mOsm/L) | E | 1,611.345 | 1,592.067 | 19.279 | 0.071 | <0.001 |
| Urine Protein (g/L) | A | 0.737 | 0.769 | -0.032 | 0.177 | <0.001 |
| Urine Protein (g/L) | B | 1.092 | 0.769 | 0.324 | 0.177 | <0.001 |
| Urine Protein (g/L) | C | 0.760 | 0.769 | -0.009 | 0.177 | <0.001 |
| Urine Protein (g/L) | D | 0.584 | 0.769 | -0.185 | 0.177 | <0.001 |
| Urine Protein (g/L) | E | 0.611 | 0.769 | -0.158 | 0.177 | <0.001 |
| Urine Total Volume Collected (mL) | A | 11.454 | 11.641 | -0.187 | 0.001 | 0.854 |
| Urine Total Volume Collected (mL) | B | 11.745 | 11.641 | 0.104 | 0.001 | 0.854 |
| Urine Total Volume Collected (mL) | C | 11.623 | 11.641 | -0.018 | 0.001 | 0.854 |
| Urine Total Volume Collected (mL) | D | 11.594 | 11.641 | -0.047 | 0.001 | 0.854 |
| Urine Total Volume Collected (mL) | E | 11.724 | 11.641 | 0.083 | 0.001 | 0.854 |
| Urine pH | A | 7.284 | 7.295 | -0.011 | 0.070 | <0.001 |
| Urine pH | B | 7.370 | 7.295 | 0.074 | 0.070 | <0.001 |
| Urine pH | C | 7.457 | 7.295 | 0.162 | 0.070 | <0.001 |
| Urine pH | D | 7.152 | 7.295 | -0.143 | 0.070 | <0.001 |
| Urine pH | E | 7.155 | 7.295 | -0.140 | 0.070 | <0.001 |

**Supplementary Table S3**. Histopathology for the high dose (90 mg/Kg) animals at 48 h post-dose

| Company | Animal number | Pathology description | Severity score |
| --- | --- | --- | --- |
| A | 21 | minimal sub-acute focal inflammation; hepatitis | 1 |
| A | 22 | minimal sub-acute focal inflammation; hepatitis | 1 |
| A | 23 | minimal sub-acute focal inflammation; hepatitis | 1 |
| A | 24 | minimal sub-acute focal inflammation; hepatitis | 1 |
| A | 25 | minimal sub-acute focal inflammation; hepatitis | 1 |
| B | 21 | moderate hepatocellular cytoplasmic vacuolation; periportal and midzonal degeneration | 3 |
| B | 22 | mild hepatocellular cytoplasmic vacuolation | 2 |
| B | 23 | marked hepatocellular cytoplasmic vacuolation | 3 |
| B | 24 | mild hepatocellular cytoplasmic vacuolation | 2 |
| B | 25 | marked hepatocellular cytoplasmic vacuolation | 3 |
| C | 24 | minimal multinuclear monofocal cells | 1 |
| C | 25 | mild multinuclear monofocal cells | 2 |
| C | 26 | minimal multinuclear monofocal cells | 1 |
| C | 27 | minimal vacuolar degeneration | 1 |
| C | 28 | minimal multinuclear monofocal cells | 1 |
| D | 21 | moderate midzonal degeneration; fatty infiltration | 4 |
| D | 22 | moderate periacinar glycogen lakes | 3 |
| D | 23 | marked periportal and midzonal degeneration; fatty infiltration | 5 |
| D | 24 | moderate midzonal degeneration; fatty infiltration | 4 |
| D | 25 | marked periportal and midzonal degeneration; fatty infiltration | 5 |
| E | 22 | mild multifocal hepatocyte necrosis | 2 |
| E | 23 | minimal multifocal portal inflammation | 1 |
| E | 21 | minimal multifocal portal inflammation | 1 |
| E | 24 | minimal multifocal hepatocyte necrosis | 2 |
| E | 25 | mild multifocal inflammation | 2 |

Scores are graded on a scale of 0-5 where 0 = no pathology; 1 = minimal (slight/barely perceptible changes or changes that are reversible without overt tissue damage); 2 = mild (clear pathological changes that are not extensive or severe); 3 = moderate pathological (pronounced abnormality, affecting >20% proportion of tissue); 4 = marked (widespread affecting >50% tissue or substantial abnormality associated with significant structural disruption); 5 = severe (extensive degenerative or necrotic abnormality >80% affected tissue).


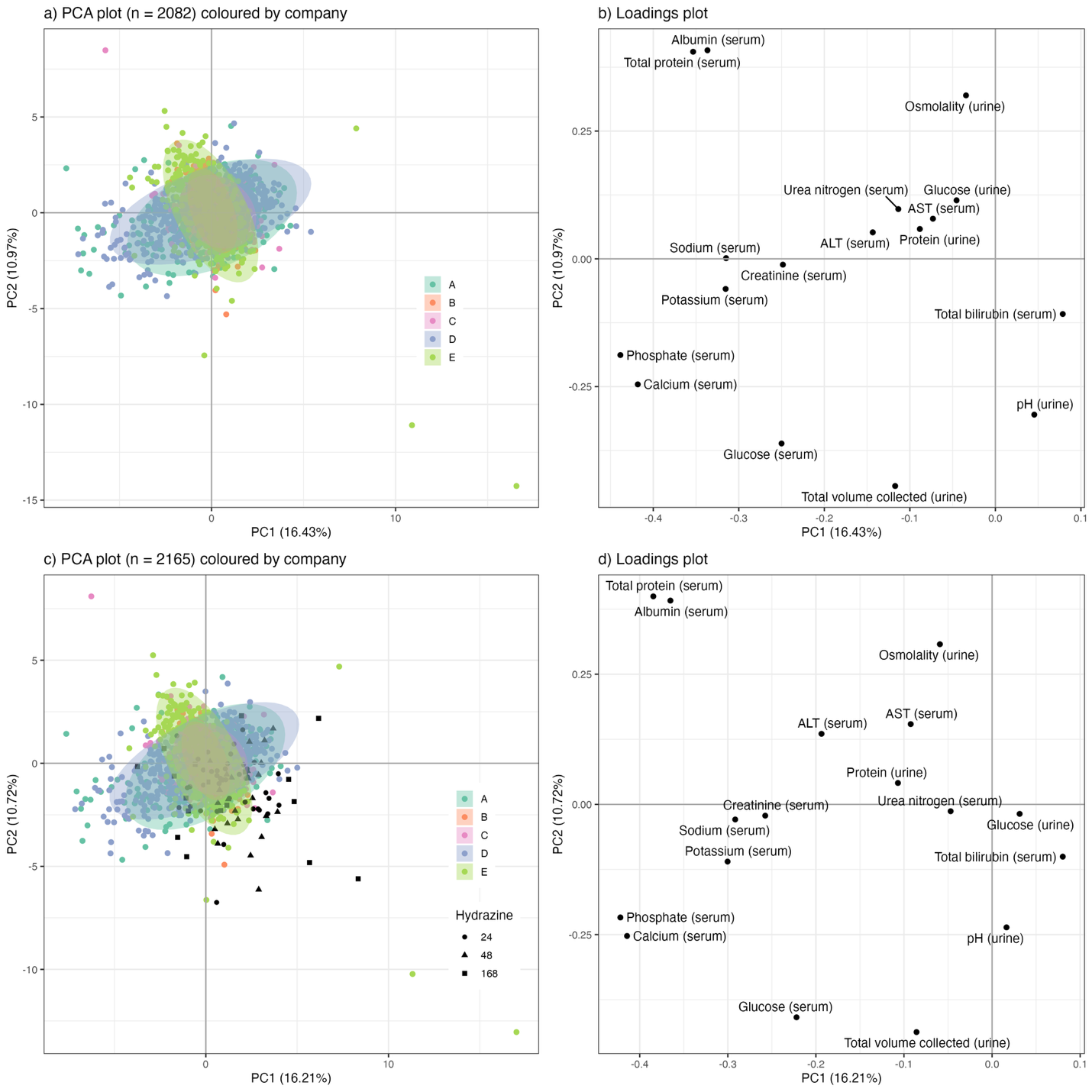


**Supplementary Fig. S2** PCA of clinical chemical parameters using control samples collected at 24 h, 48 h and 168 h post-dose from all toxin studies. Figure (a) control samples from all studies; (b) loadings plots of control samples, c) control samples from all studies and high dose hydrazine samples, d) loadings plot of control and high dose samples. PCA scores are coloured by company, and high dosed samples at 24 h, 48 and 168 hours are represented as black circles, triangles and squares respectively


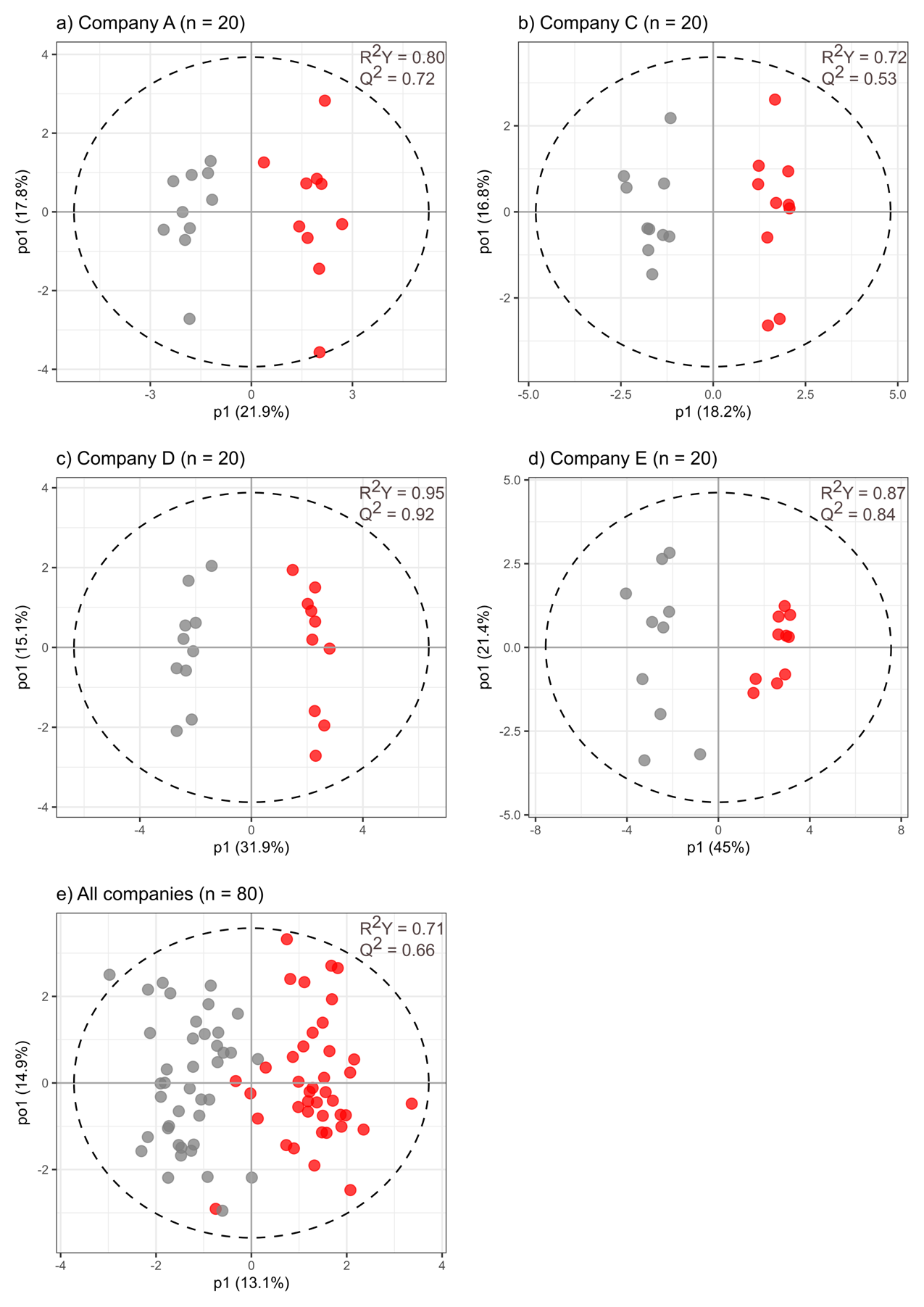


**Supplementary Figure S3.** OPLSDA scores plots comparing control (grey) and high dose (red) samples from the hydrazine studies at 24 h post-dose. Figure a - d showing scores for each individual company, figure e shows the scores for all companies combined


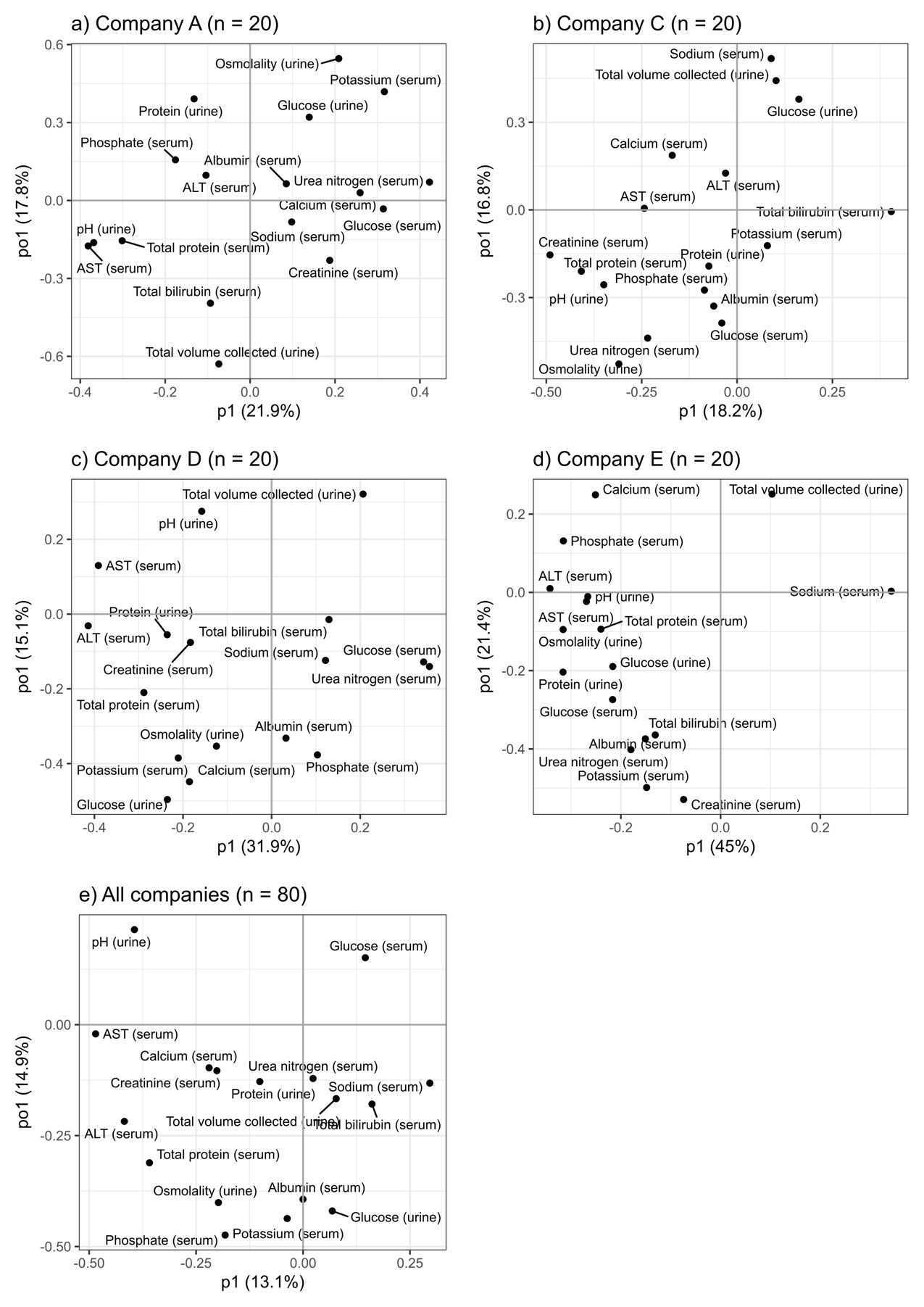


**Supplementary Figure S4.** Corresponding OPLSDA loadings plots comparing control and high dose samples from the hydrazine studies at 24 h post-dose. Figure a - d showing loadings for each individual company, figure e shows the loadings for all companies combined

​​
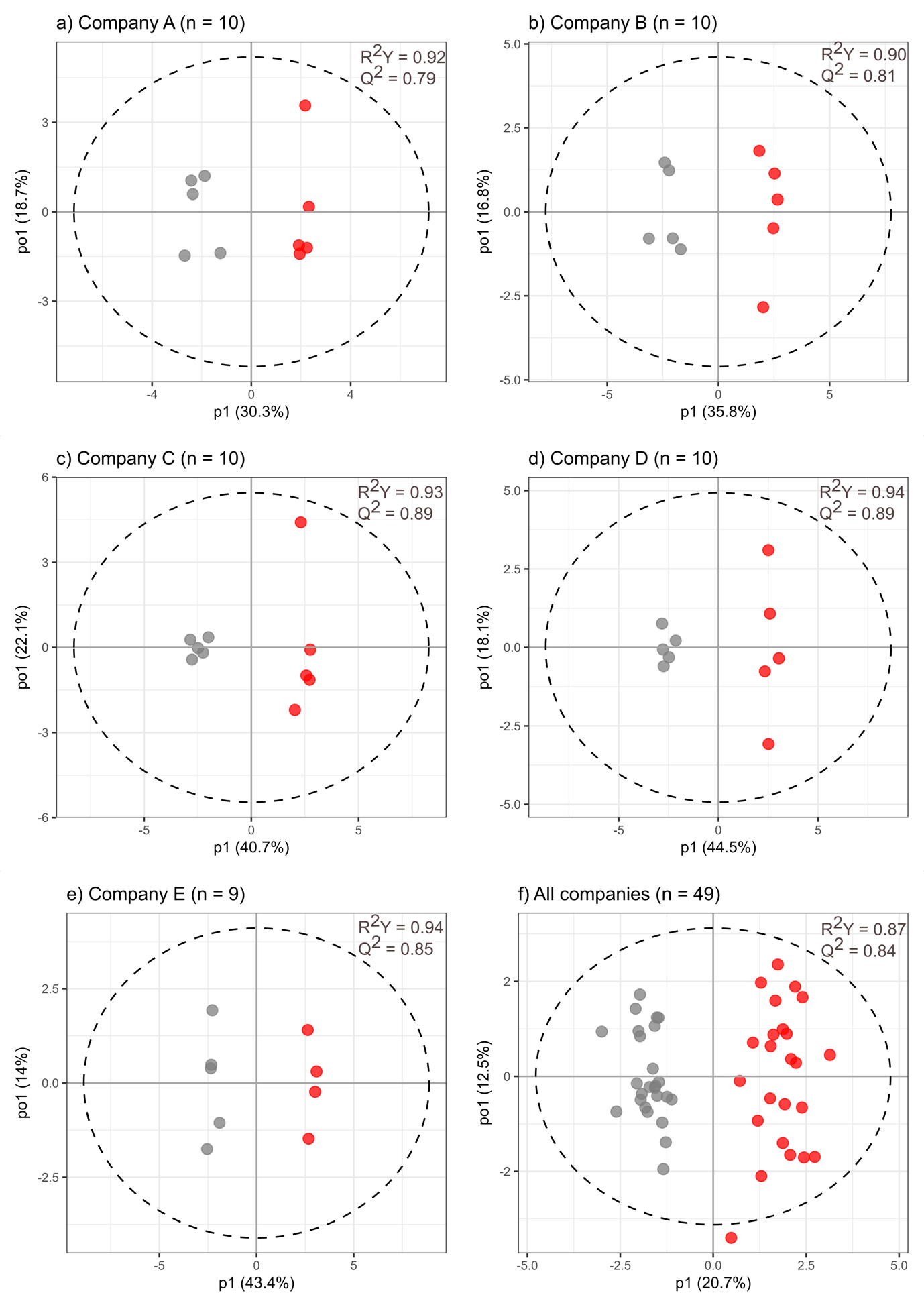


**Supplementary Figure S5.** OPLSDA scores plots comparing control (grey) and high-dose (red) samples from the hydrazine studies at 48 h post-dose. Figure a - e showing scores for each individual company, figure f shows the scores for all companies combined

**
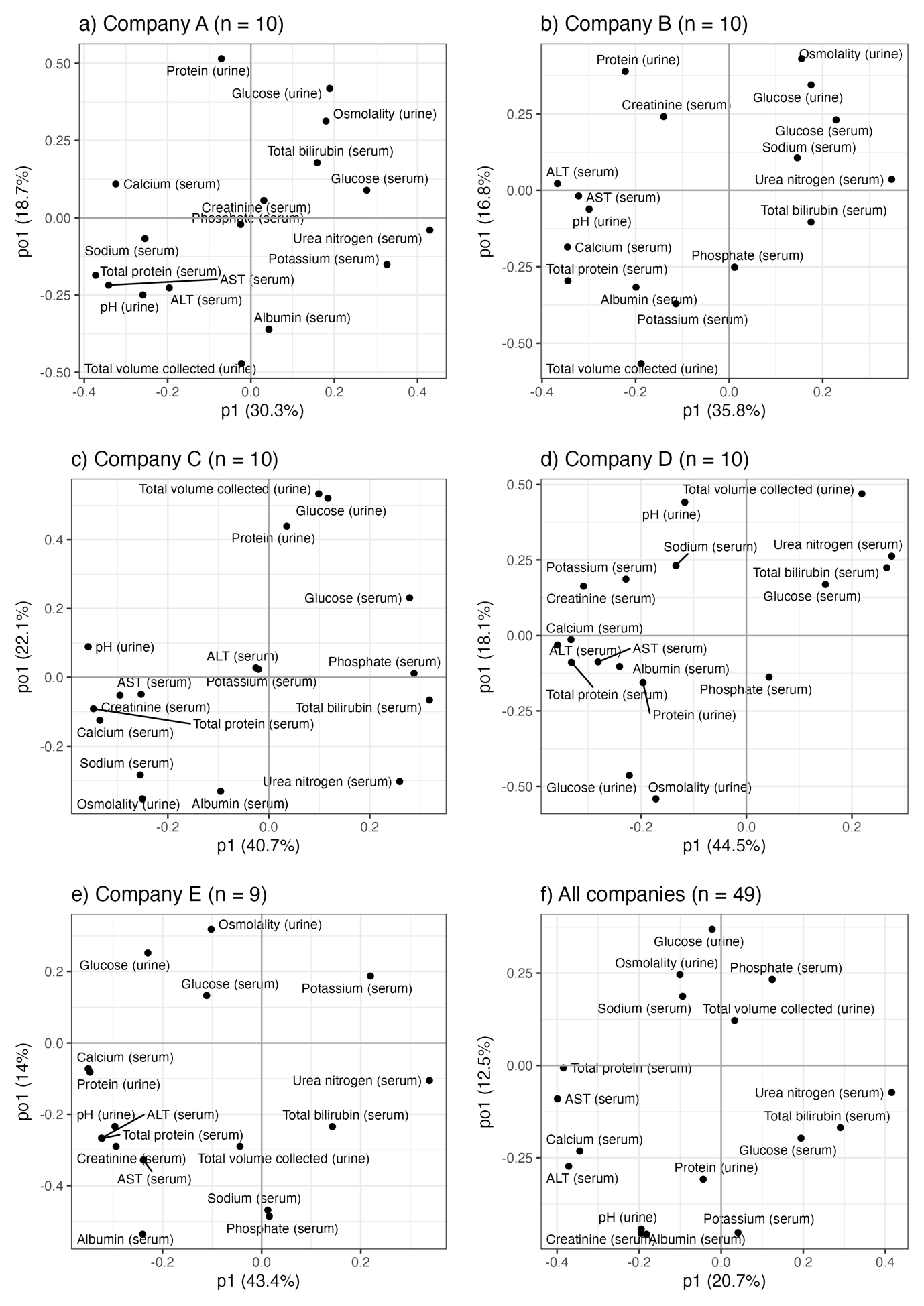
**

**Supplementary Figure S6.** Corresponding OPLSDA loadings plots comparing control and high dose samples from the hydrazine studies at 48 h post-dose. Figure a - e showing loadings for each individual company, figure f shows the loadings for all companies combined


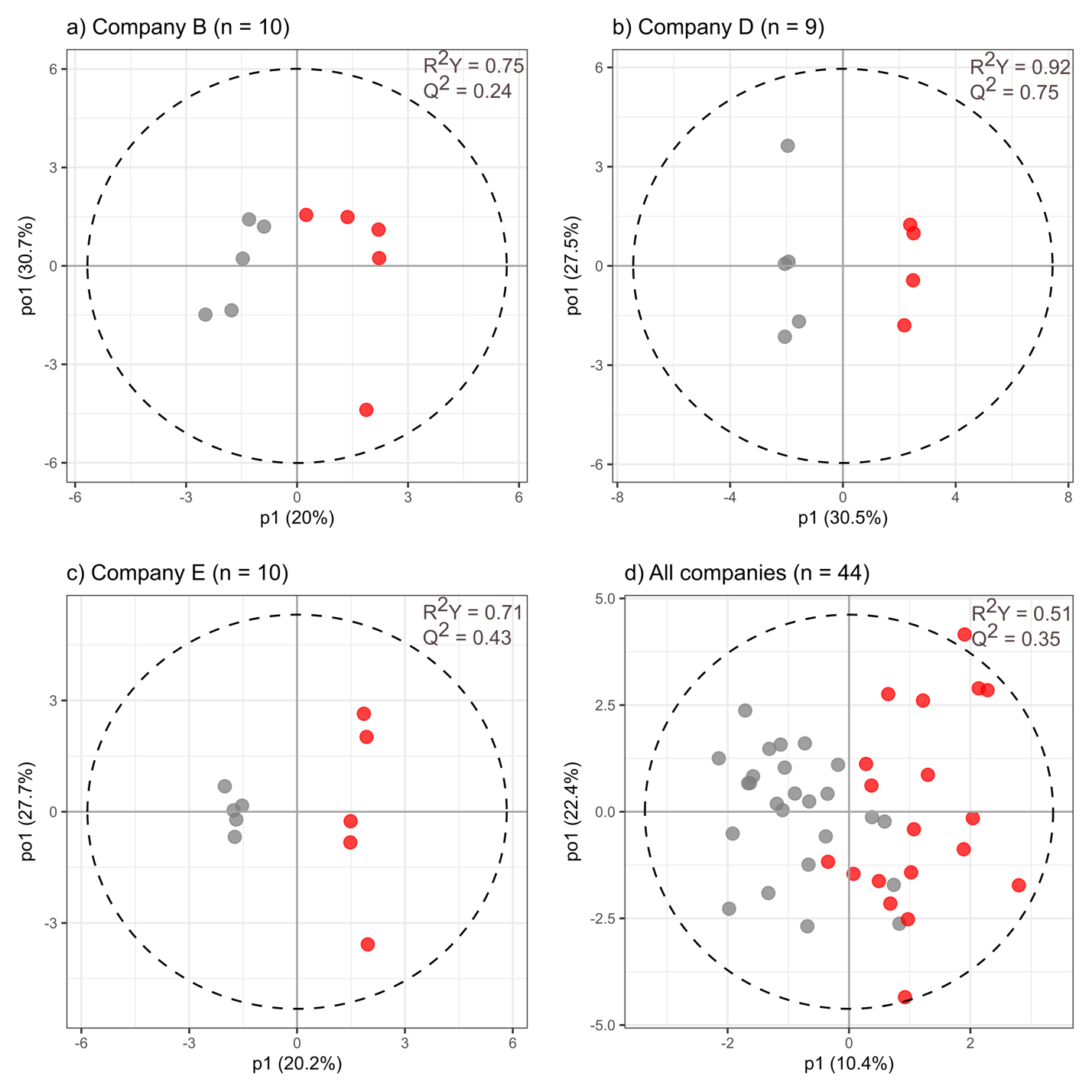


**Supplementary Figure S7.** OPLSDA scores plots comparing control (grey) and high dose (red) samples from the hydrazine studies at 168 h post-dose. Figure a - c showing scores for each individual company, figure d shows the scores for all companies combined


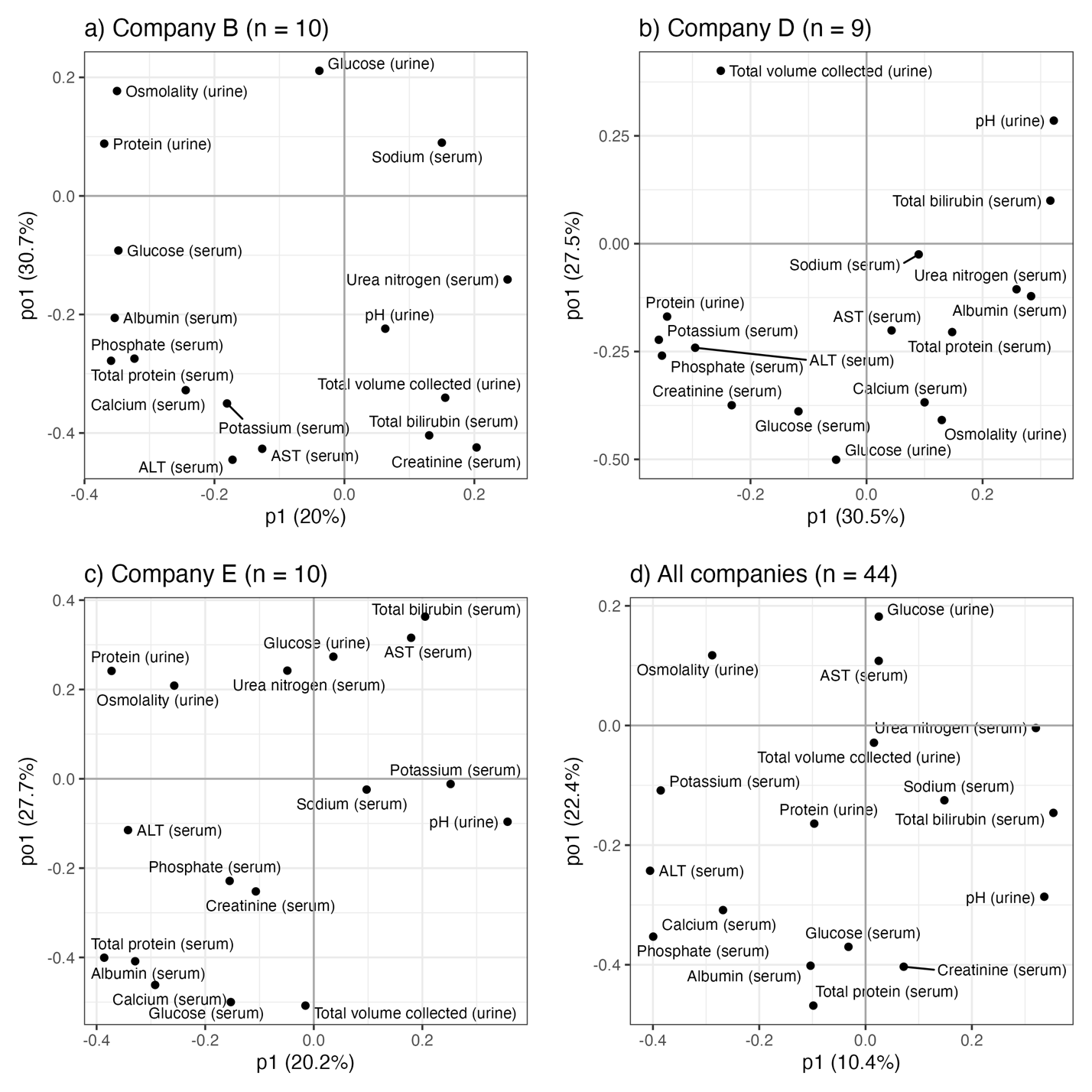


**Supplementary Figure S8.** Corresponding OPLSDA loadings plots comparing control and high dose samples from the hydrazine studies at 168 h post-dose. Figure a - c showing loadings for each individual company, figure d shows the loadings for all companies combined

**Supplementary Table S4** Top three most significant features from loadings of 18 OPLS-DA models using hydrazine study samples comparing control and high dose groups across various timepoints for individual companies and combined data. Features discriminating the control (C) group are highlighted in green, and features discriminating the high dose (HD) group are highlighted in orange. The number of times each feature appeared in the top three is listed in the last row. Strongest discriminating features for the control group are S_ALT and S_Total_protein, and for the high dose group are S_Urea_nitrogen and S_Total_bilirubin


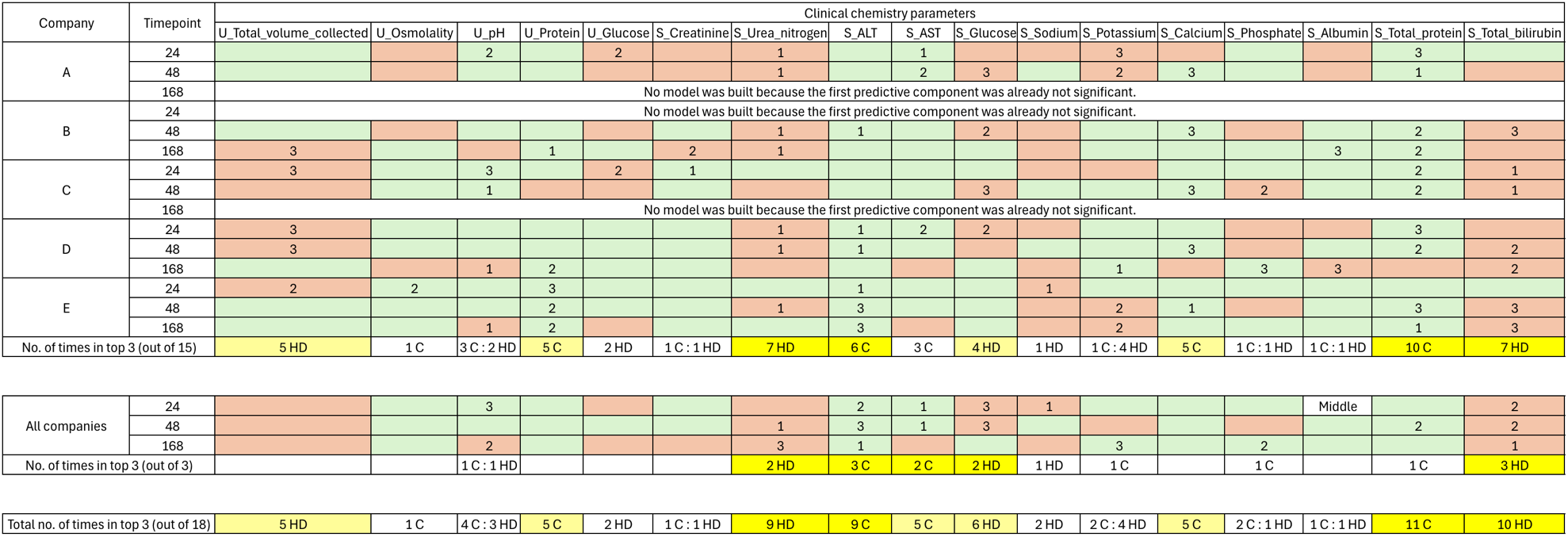


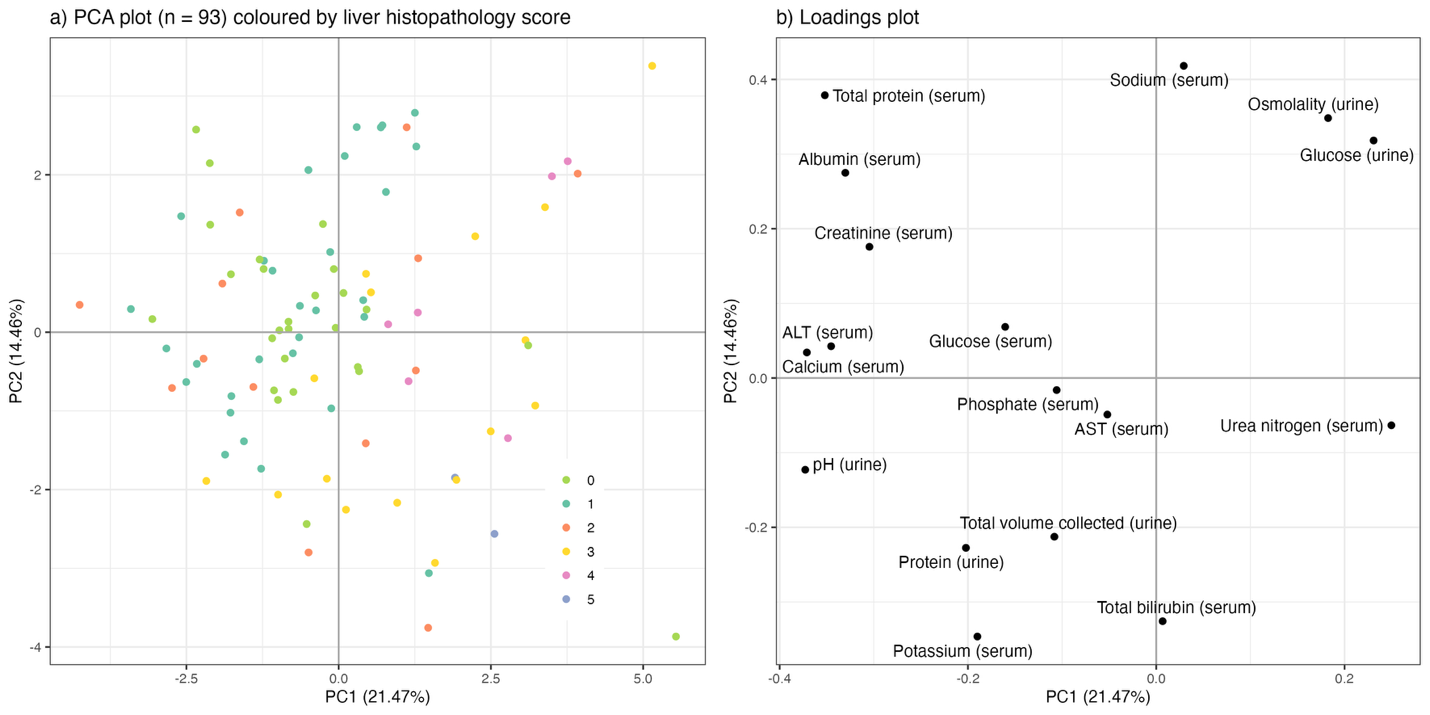


**Supplementary Figure S9.** PCA scores (a) and loadings (b) of clinical chemistry parameters using control and high dosed samples collected at 48 h and 168 h post-dose from hydrazine studies. PCA scores are coloured by liver histopathology score


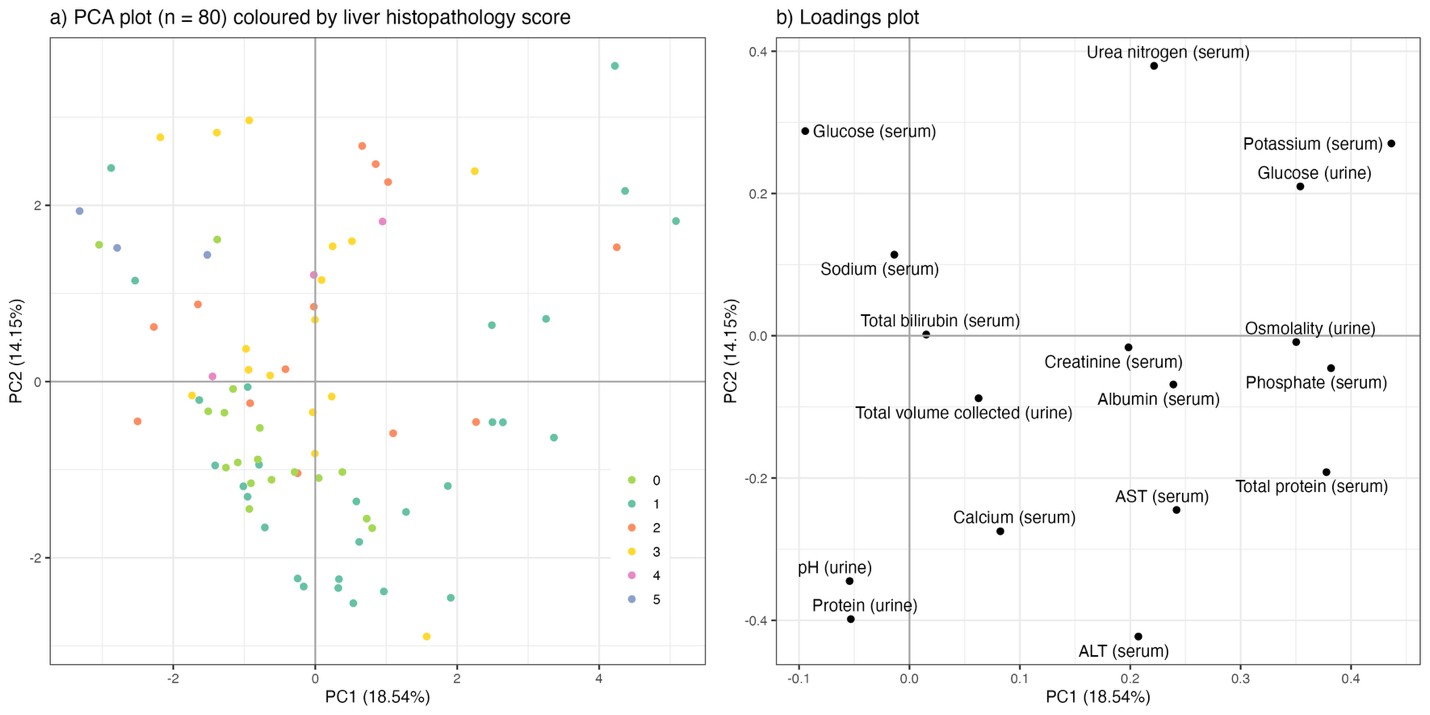


**Supplementary Figure S10.** PCA scores (a) and loadings (b) of clinical chemistry parameters using control and high dosed samples collected at 24 h post-dose from hydrazine studies. PCA scores are coloured by liver histopathology score


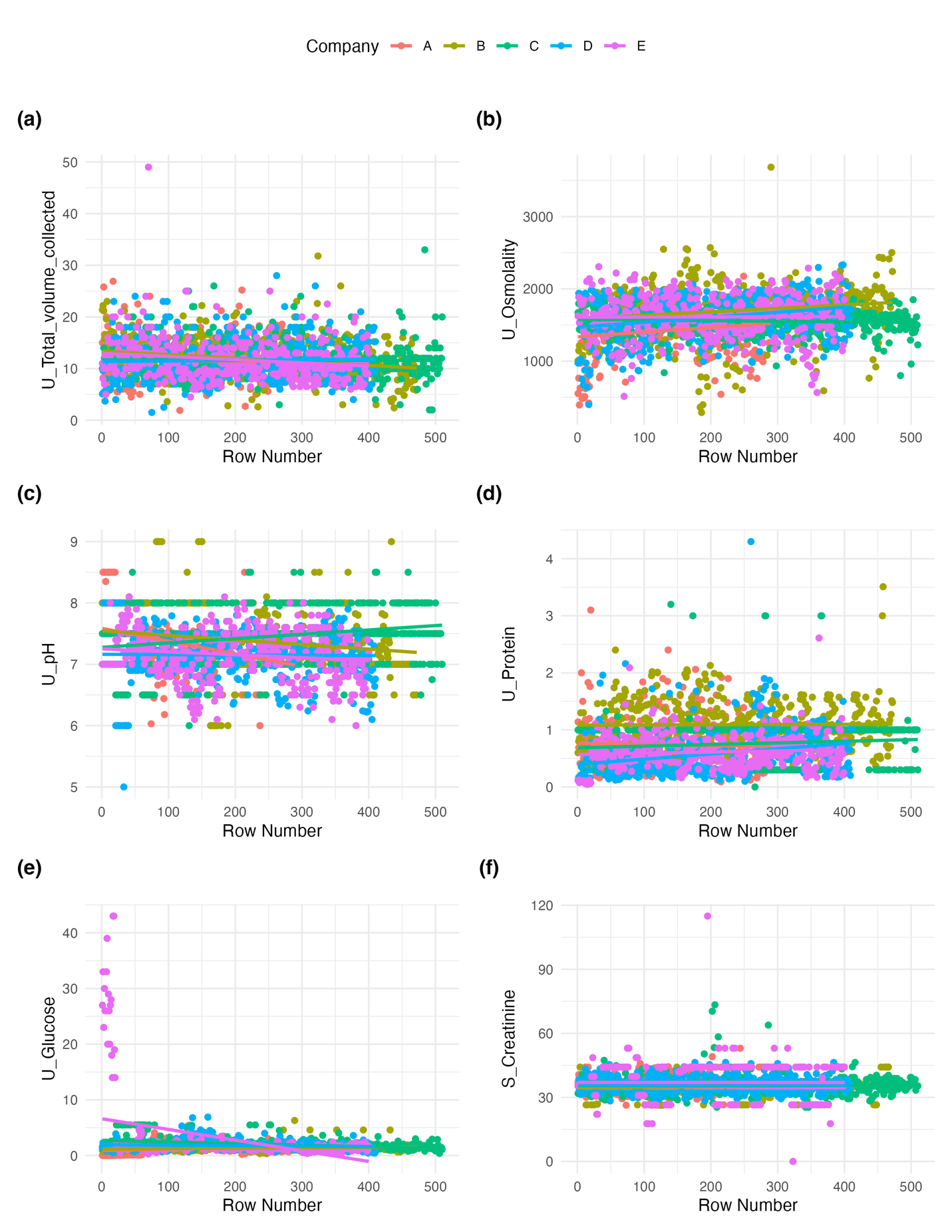


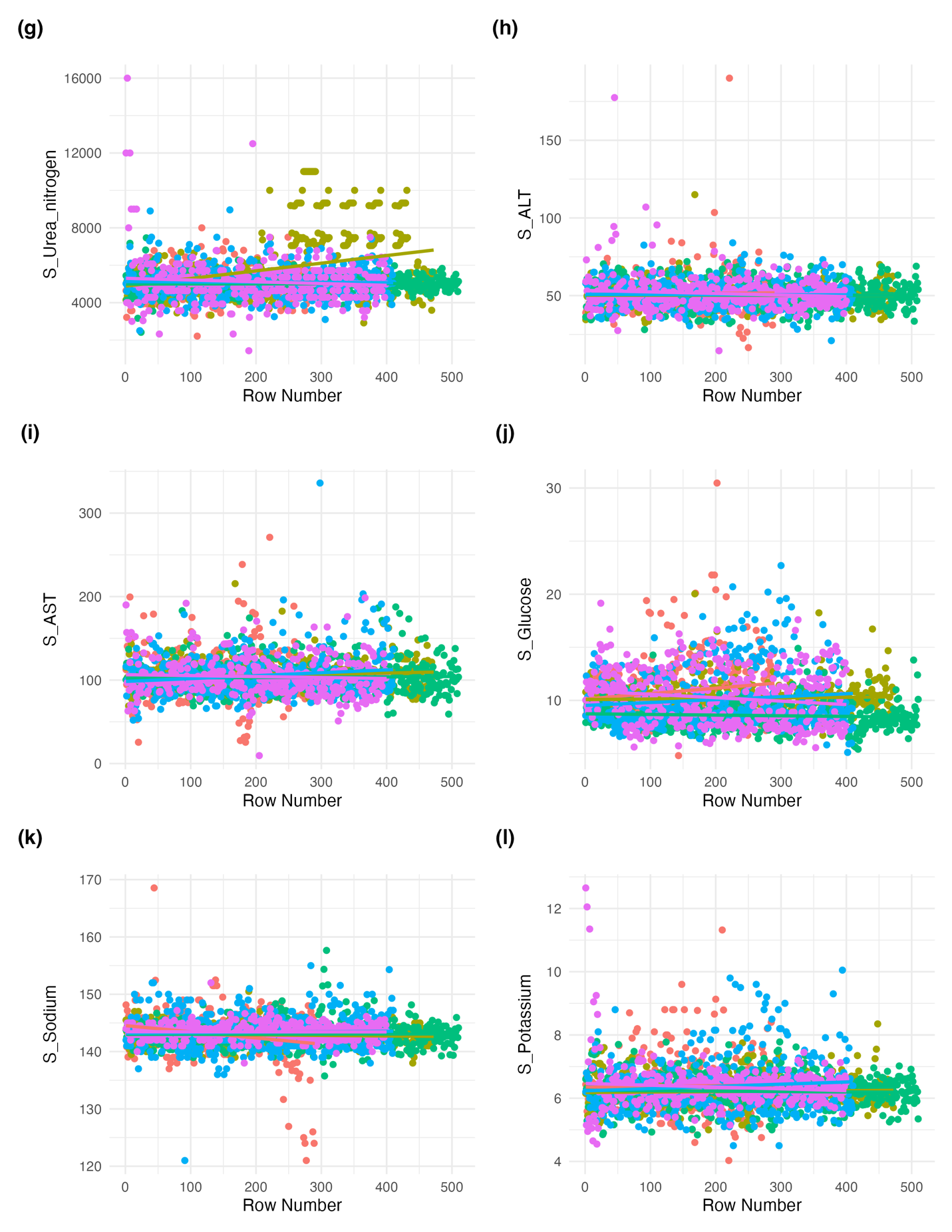


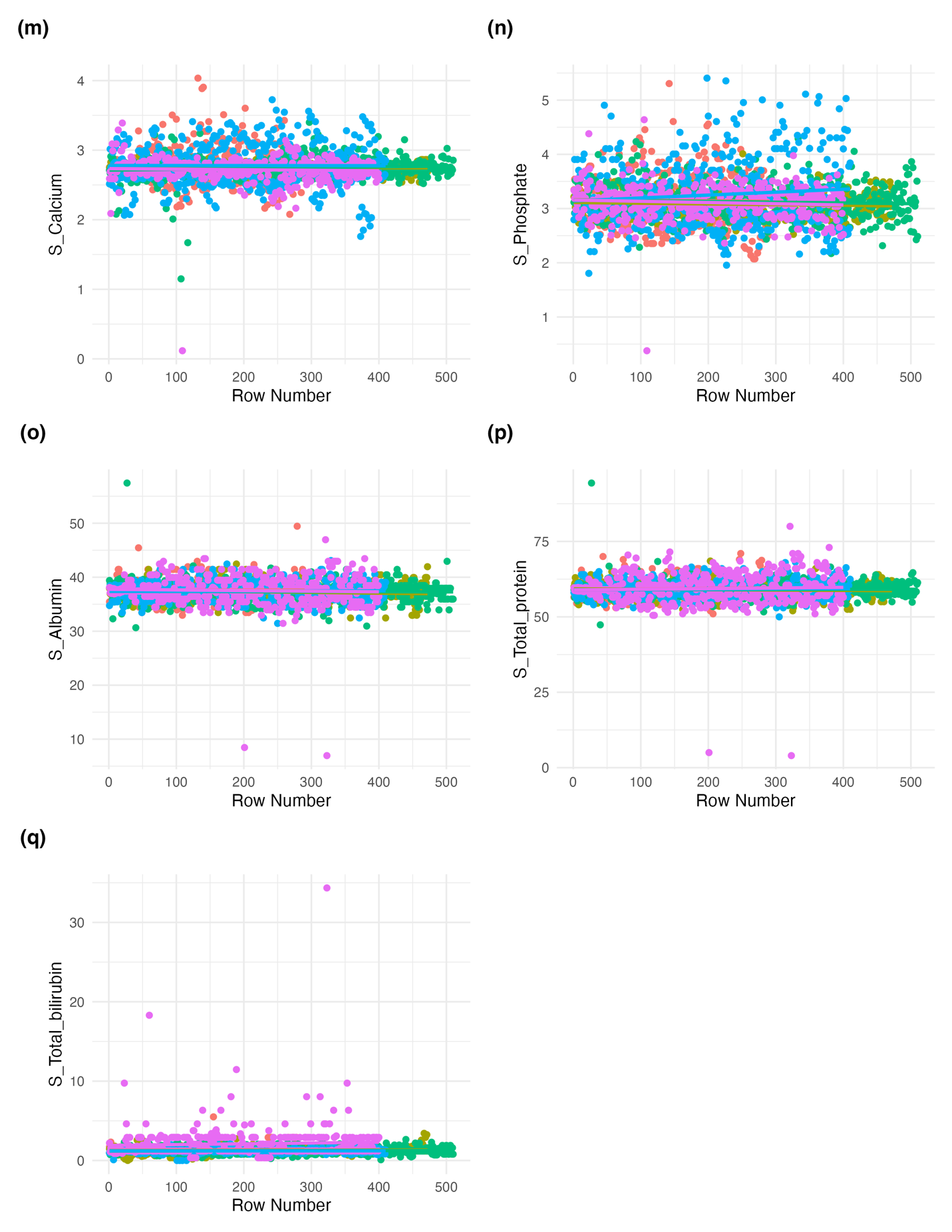


**Supplementary Figure S11.** Correlation of individual clinical chemical parameters across the three-year study duration coloured by company
